# A quantitative life history of endangered humpback chub that spawn in the Little Colorado River: variation in movement, growth, and survival

**DOI:** 10.1002/ece3.990

**Published:** 2014-02-28

**Authors:** Charles B Yackulic, Michael D Yard, Josh Korman, David R Haverbeke

**Affiliations:** 1 U.S. Geological Survey, Southwest Biological Science Center, Grand Canyon Monitoring and Research CenterFlagstaff, Arizona, 86001; 2 Ecometric Research Inc.3560 West 22nd Avenue, Vancouver, British Columbia, V6S 1J3, Canada; 3 U.S. Fish and Wildlife Service, Southwest Forest Service Science ComplexBldg 82 West, 2500 S. Pine Knoll Dr., Flagstaff, Arizona, 86001

**Keywords:** Dams, fitness trade-offs, Grand Canyon, hydrology, multistate, partial migration, size-dependent, skip spawning, tributary

## Abstract

While the ecology and evolution of partial migratory systems (defined broadly to include skip spawning) have been well studied, we are only beginning to understand how partial migratory populations are responding to ongoing environmental change. Environmental change can lead to differences in the fitness of residents and migrants, which could eventually lead to changes in the frequency of the strategies in the overall population. Here, we address questions concerning the life history of the endangered *Gila cypha* (humpback chub) in the regulated Colorado River and the unregulated tributary and primary spawning area, the Little Colorado River. We develop eight multistate models for the population based on three movement hypotheses, in which states are defined in terms of fish size classes and river locations. We fit these models to mark–recapture data collected in 2009–2012. We compare survival and growth estimates between the Colorado River and Little Colorado River and calculate abundances for all size classes. The best model supports the hypotheses that larger adults spawn more frequently than smaller adults, that there are residents in the spawning grounds, and that juveniles move out of the Little Colorado River in large numbers during the monsoon season (July–September). Monthly survival rates for *G. cypha* in the Colorado River are higher than in the Little Colorado River in all size classes; however, growth is slower. While the hypothetical life histories of life-long residents in the Little Colorado River and partial migrants spending most of its time in the Colorado River are very different, they lead to roughly similar fitness expectations when we used expected number of spawns as a proxy. However, more research is needed because our study period covers a period of years when conditions in the Colorado River for *G. cypha* are likely to have been better than has been typical over the last few decades.

## Introduction

Partial migration, in which a portion of a population is migratory and the rest is sedentary, is a widespread phenomenon observed in a variety of taxa, including fish (Jonsson and Jonsson 1993), birds (Lundberg 1988), amphibians (Grayson et al. 2011), and reptiles (Blake et al. 2013). Partial migratory systems are complex and may include individuals with movement strategies that are not easily classified. Nonetheless, three idealized forms of partial migration are recognized: (1) shared breeding (spawning) areas: residents and migrants occupy the same general area during breeding season and migrants move away from the breeding grounds during the nonbreeding season (e.g., Lack 1943), (2) shared nonbreeding areas: residents and migrants occupy the same general area during the nonbreeding season and migrants move elsewhere to breed (e.g., Morrissey et al. 2004), and (3) skip spawning: only a fraction of potential breeders leave nonbreeding areas in a given year to travel to breeding (or spawning) grounds (Shaw and Levin 2011). Although partial migration of all three types is widespread, little is known about how human modification of the environment is impacting different portions of partial migratory populations. Existing studies suggest that impacts of environmental change on partial migratory systems can be nonintuitive (Berthold 2001; Nilsson et al. 2006) and involve both positive and negative impacts on the fitness of a given strategy (Hebblewhite and Merrill 2007, 2011).

River systems are among the most modified ecosystems worldwide (Poff and Zimmerman 2010), and dams can have profound impacts on fully and partially migratory fish populations (Pringle 2003). Much research has focused on impacts dams have on connectivity in river systems such as severing some migration routes and making others more arduous (Ward and Standford 1995; Pringle 2003). In many cases, dams also change downriver environmental conditions (e.g., temperature; Olden and Naiman 2010) and are associated with changes in ecological communities, including introductions of nonnative fish (Ward and Standford 1983; Cross et al. 2013). Changes in environmental conditions impact vital rates for populations that spend some or all of their time in these waters (Poff and Hart 2002). For example, Nelson et al. (2002) concluded that the decline of the migratory contingent of partial migratory populations of *Salvelinus confluentus* (bull trout) found in the headwaters of the Bitterroot River (Montana) was not directly related to presence of the dam, but rather to changes in factors such as predation or temperature in the main river (hereafter mainstem).

**Figure 1:**
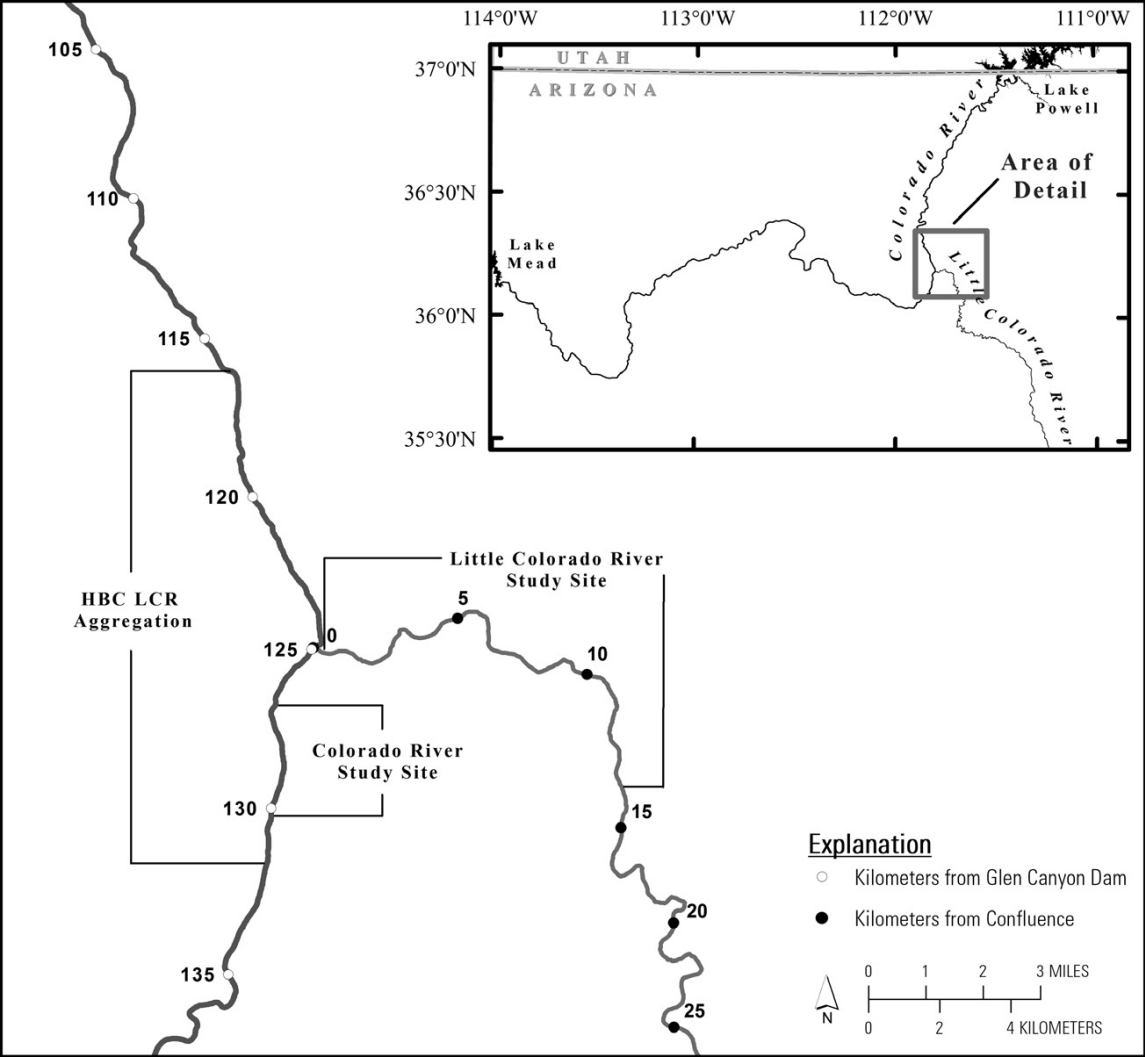
Map illustrating the spatial location of sampling in the Colorado River and Little Colorado rivers.

Prior to introduction of nonnative fishes and completion of numerous dams, federally listed *Gila cypha* (humpback chub, hereafter chub) was widely distributed in the Colorado River (USFWS 1994). Chub have since declined throughout their range and are restricted to six extant populations. The largest of these populations, and the focus of our research, is isolated from upstream populations by Glen Canyon Dam. Over 95% of this population resides in the lower 14 km of the Little Colorado River (hereafter LCR) and in the mainstem Colorado River within a few km of the LCR (Valdez and Ryel 1995; Fig. 1). Chub population trends are often attributed to conditions in the Colorado River portion of this river system, which is regulated and highly altered in terms of temperature, turbidity, and flow and which contains many introduced species, including salmonids. This population declined during the late 1990s, coincident with cooler water temperatures and higher salmonid abundances, and partially recovered over the last decade, when water temperatures were warmer and salmonid abundances were lower (Coggins et al. 2006a,2006b; Yard et al. 2011).

Chub spawn almost exclusively in the LCR during April and May. Juvenile chub that move from the LCR to the Colorado River at small sizes in May or June rarely survive (Robinson et al. 1998; Robinson and Childs 2001); however, the fate of juveniles leaving the LCR during the monsoon season between July and September appears to vary with environmental conditions (e.g., water temperature and salmonid abundances). Research during the 1990s suggested that the number of juveniles leaving the LCR between July and September can be substantial; however, very few of the fish appeared to survive (Valdez and Ryel 1995; Clarkson and Childs 2000). Given the presence of adult chub in the Colorado River, many scientists and managers in the system concluded that successful chub likely reared in the LCR before switching to a migratory life history at larger sizes. More recent studies suggest that juvenile chub can rear in both the LCR and Colorado River (Finch 2012), resurrecting basic questions about chub life history. The rate of this juvenile outmigration is unknown, as are the rates of movements between the Colorado River and LCR at later life stages. Adults that rear in the Colorado River eventually return to spawn; however, it is believed that some unknown proportion of the adult population forgoes spawning every year (Coggins et al. 2006b). In addition, Douglas and Marsh (1996) showed that adults can be found in the LCR in all months of the year and hypothesized that a portion of the adult population might reside in the LCR year-round. That is, the population may exhibit both a shared breeding partial migratory system and a skip-spawning system.

Here, we ask four general questions about chub life history. Firstly, what is the rate of skip spawning in chub and does this rate change as fish grow? A priori*,* we hypothesized that larger adults should spawn more frequently, as has been suggested for chub (Gorman and Stone 1999) and observed in other species (Secor 2007). Secondly, are there chub that reside in the LCR year-round? Thirdly, what are the rates of movement between the LCR and the Colorado River prior to adulthood? Lastly, how do survival and growth of different size classes vary between the Colorado River and the LCR and what is the fitness of migrants relative to residents? Previous work suggests that fish grow more quickly in the LCR; however, comparisons of survival rates for different portions of the river have not previously been estimable because of a lack of systematic sampling in the Colorado River. We also estimate population size for different size classes of chub, including the total adult abundance and link trends in abundance to movement hypotheses.

## Materials and Methods

### Study sites

The chub population that spawns in the Little Colorado River is primarily concentrated in the 13.6 km of the LCR that are closest to the Colorado River, and an adjacent ∼17 km section of the Colorado River referred to as the LCR aggregation (Valdez and Ryel 1995). The Colorado River is colder (average difference of 6.4°C during the study period) and ∼50 times larger than the LCR (see USGS gauges 09402500 and 09402300 for detailed discharge and temperature records). Our analysis is based on data collected in the LCR study site, which spans the lower 13.6 km of the LCR, and the Colorado River study site, which spans ∼2.7 km of the Colorado River just downriver of the confluence of the LCR and Colorado Rivers (Fig. 1 & Table 1).

**Table 1:** Approximate timing of important biological events and sampling efforts.

Month	J	F	M	A	M	J	J	A	S	O	N	D
Biological events												
Migrating chub move from Colorado River to LCR												
Chub spawn												
Most adult chub leave LCR and return to Colorado River												
Monsoon season												
Sampling efforts												
System-wide mark–recapture in LCR (2009–2012)				X	X				X	X		
Spatially restricted mark-only in LCR (2009–2011)							X	X				
Spatially restricted mark-only in LCR (2012)							X					
Sampling in Colorado River study site (2009–2011)							X	X	X	X		
Sampling in Colorado River study site (2012)				X			X		X			

Grey shading indicates the approximate timing of biological events and X's indicate months during which sampling occurred.

### Data collection

Fish in the Colorado River study site were collected using two gear types: 47–90 un-baited hoop-nets (50–60 cm diameter, 100 cm long, single 10-cm throat, made of 6-mm nylon mesh, checked daily for 8–12 days per trip) and slow-speed boat electrofishing (pulsed DC current, 15–20 amps, 200–300 volts, boat speed 7–10 sec per meter of shoreline, repeated 24–72 h apart for 3–5 total passes per trip). Sampling in the LCR consisted of mark–recapture events spanning the whole LCR study site and mark-only events over a more limited spatial extent (Table 1). Mark–recapture events in the LCR included three passes at 180 net locations using un-baited hoop-nets (for more details see Van Haverbeke et al. 2013). Mark-only events occurred between 0.8 and 1.7 km upriver of the confluence in 2009–2011 and between 1.8 and 3 km in 2012. In 2009–2011, fish were caught using 7–10 hoop-nets over the course of a few days. In 2012, sampling occurred over 4 days using 18 hoop-nets and was augmented by targeted sampling for juveniles using seines and dip nets.

Upon capture, chub were measured, scanned for prior marks, and, if appropriate, marked. All chub >100 mm total length (TL) that did not already have a 134.2 kHz passive integrated transponder (PIT) tag received a PIT tag with a unique number allowing future identification of individual fish. Juvenile chub (40–100 mm TL) received a visible implant elastomer (VIE) mark that identified the current sampling trip and spatial location. This occurred regardless of whether a fish was previously marked, so juveniles accumulate marks if they are caught on multiple trips until they grow to greater than 100 mm TL. As a consequence, each juvenile carries its capture history with it (in the form of VIE marks); however, information on individual covariates is lost. Juveniles that were captured or recaptured in the LCR study site during spring sampling received no mark, and these captures were not included in our analysis.

### Model overview

**Figure 2:**
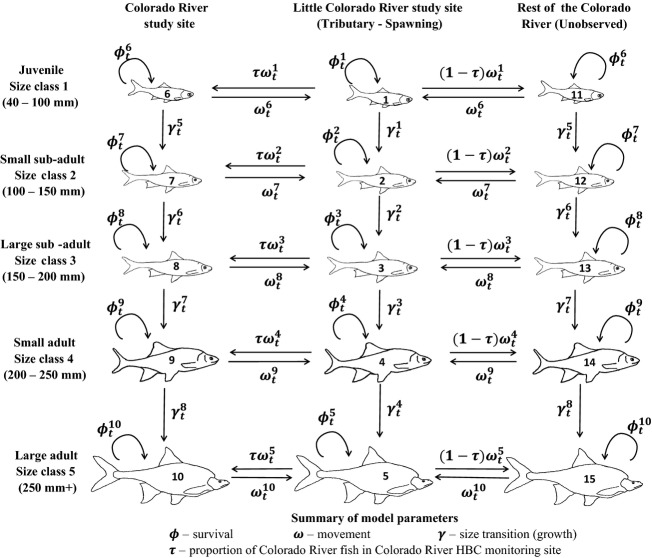
Schematic representation of the general form of the multistate model used in this paper. The number on each fish is its state.

**Figure 3:**
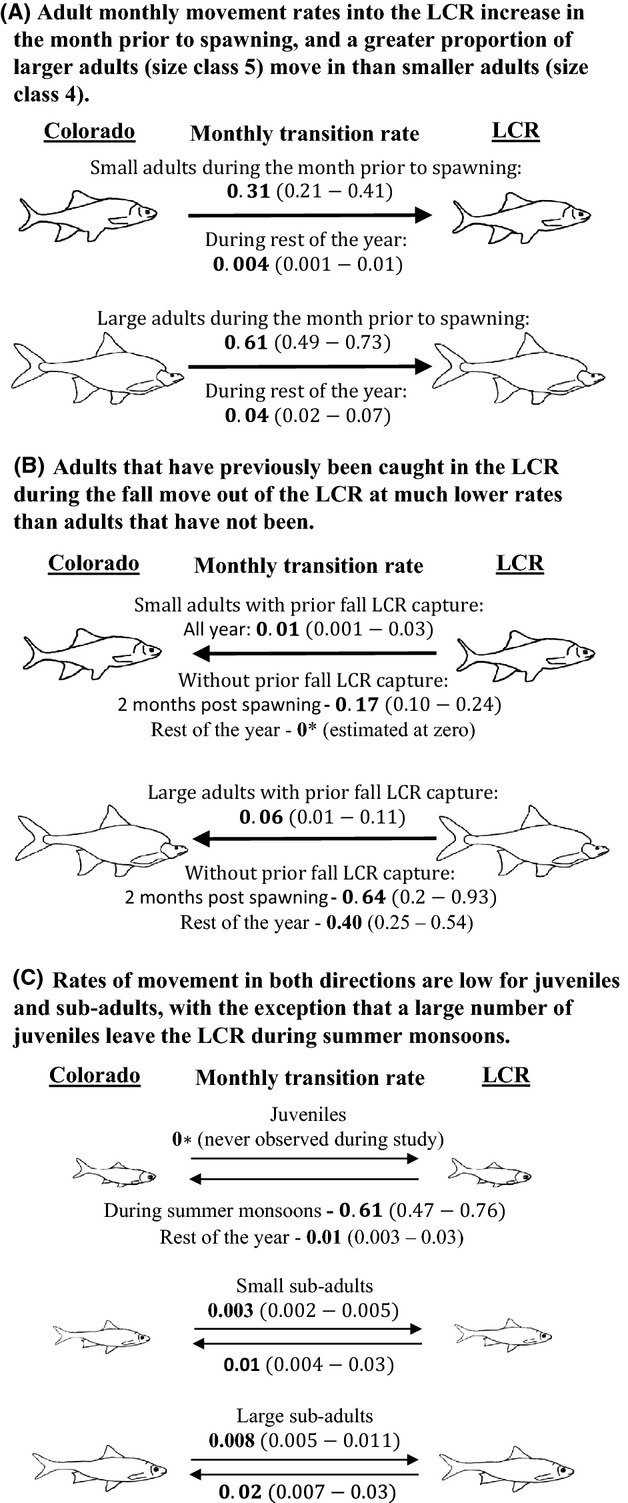
Point estimates (and 95% confidence intervals) for monthly transition probabilities associated with three movement hypotheses.

We use multistate models conditioned on first capture (Arnason 1973; Brownie et al. 1993; Schwarz et al. 1993; Lebreton et al. 2009) to test movement hypotheses and determine the relative fitness of different life-history strategies. Multistate models allow ecologists to estimate transitions between biological states (e.g., size or age classes, spatial location) when capture and/or survival probabilities vary between states and are less than one. In our models, states are defined in terms of the size of chub and their capture location and we estimate movement and growth rates, in addition to survival and capture probabilities (Fig. 2). We define five size classes and test hypotheses about the movement of juveniles (size class 1: 40–100 mm TL), smaller adults (size class 4: 200–250 mm), and larger adults (size class 5: >250 mm). We consider three locations: (1) the LCR study site, (2) the Colorado River study site (Fig. 1), and (3) the Colorado River outside of the monitoring site. States one through five correspond to the five size classes in the LCR, states six through ten correspond to the five size classes in the Colorado River study site, and states 11 through 15 correspond to the five size classes elsewhere in the Colorado River (Fig. 2). Inclusion of states 11 through 15 allows us to account for fish in the Colorado River outside of the Colorado River study site that temporally emigrate into the LCR to spawn some years, but are otherwise unavailable for capture. Chub vital rates (growth, survival, etc.) for a given size class in the Colorado River study site are assumed to be representative of vital rates for all chub in that size class in the Colorado River (Fig. 2).

Our study period is from April 2009 and October 2012, and we assume a monthly time step for modeling. Of a possible 43 months, the Colorado River study site is sampled 15 times and the LCR is sampled 23 times (Table 1). For computational reasons (our data include 30,067 individual capture histories) and because we did not seek to fit models with between-trip trap responses or individual variation, we decomposed capture histories into a series of release–recapture events and determined the frequency of these events (sensu Pradel 1993). In other words, we summarized the information in our individual capture histories in an 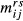 array (Williams et al. 2002, p. 456) and analyzed the information contained in this array.

### Survival and state dynamics

In our model survival, 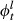 is defined as the probability that a marked fish in state *l* at time *t* survives from month *t* to *t + *1, regardless of whether the individual transitions into another state during the same interval. All the models examined in this paper assume that survival is constant through time (i.e., 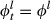), but allow survival to vary between states (with the exception that fish in the same size class in the Colorado River have the same survival regardless of whether they are in the Colorado River study site or elsewhere). Our code is written to allow future examination of variation in survival over time (Appendix S1). State dynamics (i.e., movement and growth) were characterized generally using state transition parameters, 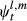, reflecting the probability that a fish in state *l* at time *t* will transition to state *m* at time *t + *1, conditional on survival until *t *+* *1. For the purposes of our study, transition probabilities are expressed in terms of (1) growth conditional on survival and (2) movement conditional on survival and growth. Growth was assumed to be constant over time, to vary between those states in which growth was possible (i.e.*,* it is not possible to transition out of states associated with size class 5), and to be the same for a given size class found in either of the two Colorado River locations. As such, we considered eight growth probabilities, *γ*^*i*^, where the first four parameters (*i = *1,2,3, or 4) correspond to the probability of transitioning out of size classes one through four in the LCR, and the next four (*i = *5,6,7, or 8) are defined as the probabilities of growing out of size classes one through four in the Colorado River.

Movement probabilities were constrained based on two assumptions. First, we assumed that movement of chub between the Colorado River study site and other parts of the Colorado River is negligible. We base this assumption on studies over both short and long temporal scales that suggest that chub exhibit strong patterns of spatial fidelity (outside of migration) when they are in the Colorado River, restricting their movements to an average linear extent of around 500 m (Valdez and Ryel 1995; Paukert et al. 2006; Gerig 2012). Second, we assumed that a constant proportion, *τ*, of chub within the Colorado River are found within the Colorado River study site, regardless of size class. This assumption should be approximately true provided that vital rates in the Colorado River study site are similar to elsewhere in the Colorado River. Based on these assumptions, we decomposed movement in a vector or probabilities, 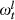, representing the probability of a state transition corresponding to moving into the same size class in the other part of the river system (i.e., moving in the Colorado River if currently in the LCR or vice-versa) between time *t* and *t + *1. For chub moving from the LCR into the Colorado River, 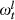 was multiplied by *τ* to express movement into the Colorado River study site and multiplied by (1 − *τ*) for movement into areas outside of the Colorado River study site. For states associated with size classes 2 and 3 (i.e., *l *=* *2, 3, 7, 8, 12, or 13), 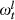 was assumed to be constant through time; however, 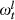 varied by month (and depending on prior capture history) for other size classes based on a priori hypotheses (see below). As with growth and survival, movement probabilities out of the Colorado River were assumed to be the same regardless of whether a fish was in the Colorado River study site or elsewhere in the Colorado (e.g., *ω*^7^ = *ω*^12^). Lastly, because no instances of juvenile fish moving from the Colorado River to the LCR were observed in our data set, the associated parameters (*ω*^6^) were fixed at zero, for all *t*.

We incorporated the joint effects of survival and transition probabilities in an array, 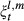, to express the probability that an individual in state *l* at time *t* both survives and transitions to state *m* in time *t + *1. For example, the probability of moving from state 1 (juvenile in LCR) to state 6 (juvenile in the Colorado River study site) can be expressed as follows:


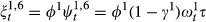
(1)

which states that the probability of surviving and moving from state 1 to state 6 is the product of the probability of surviving in state 1 (*φ*^1^), the probability of not growing into state 2 (1−*γ*^1^), the probability of moving from the LCR into the Colorado River 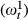, and the probability of ending in the Colorado River study site (*τ*).

### Capture probability and tag loss

We assume that when states were observed, they were observed perfectly (i.e., fish were assigned to the correct size class). We account for the possibility that chub could be in a particular state and be unobserved using a vector of parameters, 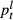, indicating the probability that a fish in true state *l* during time *t* is captured. All of the models we fit estimate capture probability separately for each state and time period in which there was a possibility of recapture. As states 11 through 15 are never directly observed, capture probabilities for these states are always set to zero. To account for tagging mortality and tag-shedding rates, we incorporated additional parameters into our models and fixed them at values estimated from laboratory experiments on sister taxa (*Gila elegans* and *Gila intermedia*; Appendix S2; Ward et al. 2008). For both PIT and VIE marks, the instantaneous rate, *δ*, of tag mortality and tag shedding was estimated at 3%. This rate represents mortality and tag loss within a few days of tagging and was only applied once for newly tagged fish. In addition, as our laboratory studies (Appendix S2) show that VIE loss rate is higher for individuals growing quickly in warmer water, we also include a monthly mark loss rate (*π*) of 0.03 for VIE tagged fish in the LCR where juvenile growth rates are higher.

### A priori hypotheses

We evaluate a set of eight models to address three movement hypotheses (Table 2). *Hypothesis* 1 *(H*1*):* Larger adults spawn more frequently than smaller adults, so chub in state nine (size class four) will have lower movement rates (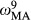 – where the subscript refers to the interval between March and April) from the Colorado River to the LCR between March and April than chub in state ten (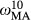). *H2:* Adult chub caught in the LCR during the fall may be residents (*R*) and thus more likely to remain in the LCR in the future than other adults (likely migrants, *M*). In other words, movement of potential adult residents out of the LCR (*ω*^4*R*^ and *ω*^5*R*^ if rates are estimated separately for smaller and larger adults) will be less than movements of migrants, especially in the 2 months after spawning (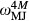 and 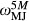 – where the subscripts refer to the period between May and June). *H3:* There is a large outmigration of juvenile fish from the LCR during the monsoon season in their first year of life, so models that estimate a different rate of movement out of the LCR for juvenile chub between July and September, 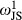, than during the rest of the year, 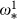, will fit the data better than a model that assumes a constant movement rate, *ω*^1^, throughout the year. Although we considered models that allowed for variation between months according to our a priori hypotheses, all models assumed that there was no interannual variation in rates.

**Table 2:** Model selection results. For all models, capture probability is allowed to vary for each state and sampling period.

Model Number	Estimated movement parameters that differed between models (in addition to ω^2^, ω^3^, ω^7^, ω^8^, which were estimated in all models)^1^	Different movement rates for smaller and larger adults?	Different movement rates for adults previously caught in the LCR in fall (i.e., LCR residency)?	Different rates of juvenile movement out of LCR during monsoon season?	*K*^2^	ΔQAIC^3^
1	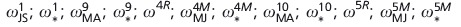	Y	Y	Y	178	0
2	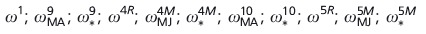	Y	Y	N	177	58
3	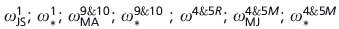	N	Y	Y	173	91
4	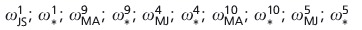	Y	N	Y	176	126
5	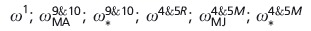	N	Y	N	172	153
6	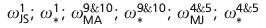	N	N	Y	172	168
7	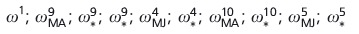	Y	N	N	175	186
8	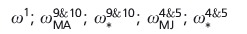	N	N	N	171	228

1 The subscript for each *ω* refers to the between month intervals for which the parameter was estimated and the superscript refers to the state from which movement occurred. Codes in the subscript are as follows: MA – March to April; MJ – May to July; JS – July to September. A *ω* without a subscript indicates that the parameters was constant across all months, while an ^*^ in the subscript indicates that a parameter was estimated for all other intervals besides the interval(s) with a special parameter for that state. Superscripts that include two states separated by an “&” indicate that this parameter is restricted to be the same for both of the states. Superscripts including a letter refer to a separate parameter being estimated for fish with (“R” – resident) and without (“M” – migrant) a prior capture during a fall LCR sampling event.

2 *K* is the total number of estimated parameters in the model.

3 ΔQAIC is the difference in quasi-likelihood between each model and the best overall model in the model set.

In addition to addressing the three movement hypotheses via model comparison, we are also interested in the degree to which estimates of chub survival and growth for different size classes vary between the Colorado River and the LCR. A priori, we hypothesized that growth rates would be lower in the Colorado River than in the LCR when comparing within a size class and that growth rates would decline for larger size classes. We also hypothesized that survival would increase for larger size classes, but had no a priori hypotheses about how survival would compare between the Colorado River and the LCR. Lastly, we hypothesized that *τ* would have a value of 0.15–0.25 based on past studies suggesting that most chub in the Colorado River are found near to the LCR confluence.

Models are fitted by maximum likelihood using *R*. Overdispersion was estimated using the 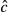 statistic computed using a scaled Pearson's chi-squared test statistic (Burnham and Anderson 1998, page 68). We compared models using quasi-AIC and used 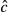 to adjust variance estimates for parameters.

### Derived parameters

To illustrate the consequences of the differences in survival and growth rates between the Colorado River and LCR, we calculate a number of derived parameters based on the maximum-likelihood estimates of survival and size transition rates and assuming that fish strictly adhere to either a skip-spawning migratory or breeding area resident strategy. In presenting these analyses, we acknowledge that some fish do not fall neatly into either category, that the period of our monitoring almost certainly does not reflect the likely variation in vital rates, and that we are ignoring various sources of uncertainty. The specific parameters we derive are the expected number of months spent in each size class, the probability of surviving from July of a fish's first year to the smaller mature size class (size class 4), the probability of surviving from July of a fish's first year to the larger mature size class (size class 5), and the expected life span of size class 5 chub conditional on them having survived to that size (and in the case of migratory fish, depending on whether they spawn every year, skip spawn, or never spawn). As a measure of the relative fitness of each strategy, we also derive the expected number of spawns as a large adult for a fish that survives to July of its first year and then adopts either the migratory or the resident strategy by multiplying the probability of surviving to size class 5 under each strategy, by the expected number of spawns after reach size class 5. We also derived estimates of abundance for different states and for the overall adult abundance using the Horvitz–Thompson estimator (see Appendix S3 for details).

## Results

The best model according to QAIC supported all three movement hypotheses (Table 2) and had a 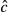 of 1.7, indicating slight overdispersion (Appendix S4). The best model suggests that some adults skip spawn and larger adults skip less frequently. In others words, larger adults have a much greater probability than smaller adults of moving into the LCR from the Colorado River in the month prior to spawning (0.61 vs. 0.31; Fig 3A). In addition, adults of both size classes that have been caught in the LCR during any prior fall (i.e., potential residents) have a smaller chance of moving out of the LCR into the Colorado River than do adult chub that have never been caught in the LCR during the fall (Fig. 3B). Lastly, the monthly probabilities of moving between locations for subadults and juveniles are generally low (Fig. 3C). The lone exception is that a large proportion of juveniles emigrate from the LCR during the monsoon season.

### Survival and size transition rates

**Figure 4:**
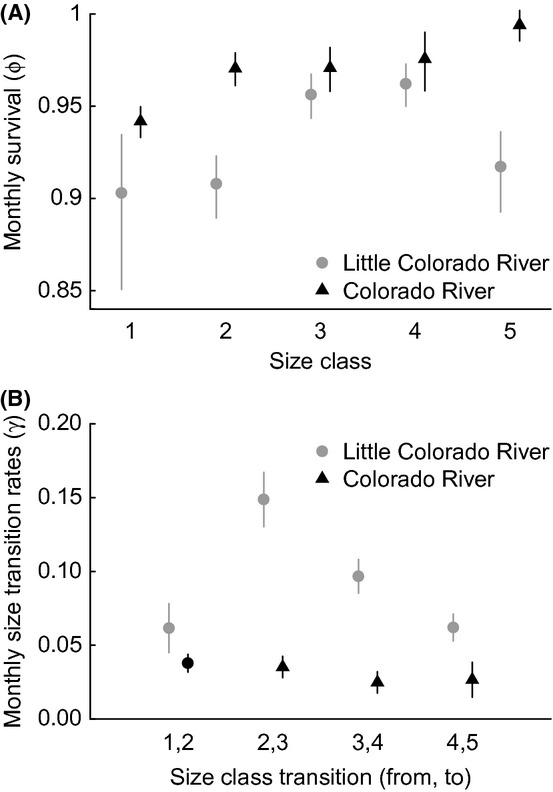
Estimates of (A) monthly survival and (B) monthly size transition rates from best model (whiskers around dots indicate 95% confidence intervals adjusted by 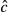).

**Figure 5:**
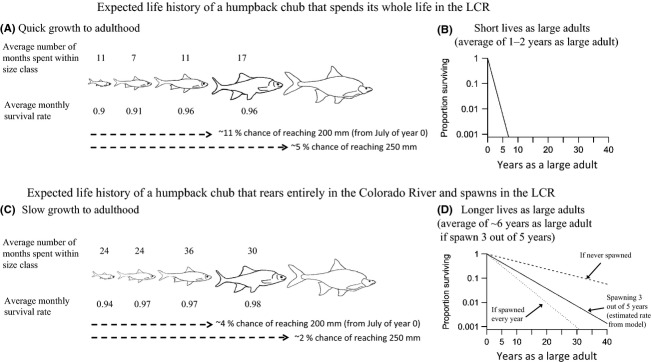
Comparison of two different strategies with similar overall fitness's. Chub that rear strictly in the LCR (A) mature quickly and (B) do not live long as adults. Chub that rear strictly in the Colorado River and then skip spawn (C) mature slowly, but (D) have much longer expected adult life spans.

Monthly survival rates are higher in the Colorado River than in the LCR for all size classes (Fig. 4A). In addition, survival generally increases for larger fish, with the exception of survival for size class 5 fish in the LCR, which is estimated as lower than survival of size classes 3 and 4. On the other hand, size transition rates are much higher in the LCR than in the Colorado River (Fig. 4B). To illustrate the potential range of life histories that these survival and size transition rates suggest, we consider two scenarios that bracket the potential variation: (1) life-long residency in the LCR starting in July of a chub's first year or (2) rearing in the Colorado River from July of the first year (and returning to the LCR only to spawn). Under the first scenario, chub quickly grow to adulthood and have short life expectancies as adults (Fig. 5). On other hand, chub that rear in the Colorado River take more than twice as long to reach adulthood and are ∼40% less likely to survive to be either small or large adults, but live ∼5 times longer, on average, as larger adults (assuming they reach adulthood). Under both scenarios, the expected number of spawns as a large adult (a proxy for fitness) is around 0.05–0.10.

The best model estimates *τ* as 0.18 (95% CI: 0.13–0.24), within the range we predicted a priori. Derived estimates of juvenile abundance in the Colorado River study site increased each year between July and September consistent with our juvenile movement hypothesis (Fig. 3C & Appendix S3). Derived estimates of other size classes and the overall adult population in the system were relatively stable over the study period, with the most precise estimate of total adult abundance coming in September 2011 (11,000 95% CI: 7000–16,000; Appendix S3).

## Discussion

Our results are consistent with the existence of both shared breeding and skip-spawning partial migration in the chub population that spawns in the LCR (Table 2; Fig. 3). While skip spawning is known to occur in this population (Coggins et al. 2006b), we provide the first estimates of rates and show that larger adults in the Colorado River migrate into the LCR during the spawning season more frequently than smaller adults, consistent with theoretical expectations and observations in most other systems (Jørgensen et al. 2006). We also found that adults that were captured in the LCR during the nonspawning season are less likely to leave the LCR at any point in the rest of the study than adult chub that have not been captured there during the nonspawning season. This observation supports the hypothesis that some adult chub reside in the LCR year-round.

Partial migration is believed to be relatively common, yet understudied, and we are only beginning to understand how humans are affecting these systems (Chapman et al. 2011). Here, we have shown that there can be large differences in vital rates within different parts of the river system that includes both unregulated and regulated habitats, and these differences in vital rates can lead to very different life histories for individuals that adopt a strictly migratory or strictly breeding ground resident strategy (Fig. 5). Despite the large differences in growth and survival between the Colorado River and the LCR, the fitness of the resident and migrant strategies may be roughly equal. For example, if we chose the number of spawns as a large adult as a proxy for the fitness of a given strategy, then expected fitness for a juvenile in July does not differ significantly based on whether it resides in the LCR for the rest of its life (0.05–0.1 expected spawns based on the probability of reaching large adulthood and life expectancy as a large adult: Fig 5A–B) or moves into the Colorado River and rears until adopting a migratory strategy as an adult (∼0.07 expected spawns based on 0.02 probability of reaching large adulthood, the expected life span of 6 years for a skip-spawning adult, and spawning rate of 0.6: Fig. 5C–D). On the other hand, the generation time of residents is roughly half that of migrants, which may mean both quicker declines and recoveries of residents relative to migrants.

While intriguing, these calculations are nonetheless based on a short period of time (3.5 years for a species that can live to be 30 + years), ignore substantial uncertainty, and occurred during a period when conditions in the Colorado River were favorable. For example, average water temperatures in the regulated Colorado River were ∼1°C colder during the 1980s through early 2000s as compared to our study period (and presumably chub growth rates were likewise lower). Furthermore, salmonid abundances near the confluence were relatively low during the study period compared with estimates from other times in the last few decades (suggesting that HBC survival rates in the Colorado River may have been substantially lower during periods of time in the past; Yard et al. 2011; Korman et al. 2012). It is impossible to determine how present variation in chub growth and survival in the Colorado River compares to the conditions over evolutionary time periods given changes in many factors expected to affect these rates including introduction of nonnative fish species, extirpation of native fish species, shifts in the food base and massive changes in the physical template (especially temperature, flow and turbidity).

While the regulated Colorado River has changed drastically over the last century, the LCR is also modified. From the perspective of chub, the greatest modification has been the addition of nonnative species that prey on humpback chub (Marsh and Douglas 1997). Determining abundances of many nonnative species in the LCR is difficult because they are not easily captured using hoop-nets. The impacts of these nonnative species on chub survival are unknown, and it has been suggested that they could be significant, particularly with respect to smaller size classes. While we had no a priori hypotheses concerning differences in survival rates between the LCR and the Colorado River, we were surprised that monthly survival rates were consistently lower among all size classes in the LCR. In particular, the apparent decline in estimates of survival between size class 4 and size class 5 fish in the LCR was unexpected and will be a focus of future research. One *a posteriori* hypothesis is that larger adults in the LCR are experiencing increased mortality associated with the physiological costs of spawning, while a second *a posteriori* hypothesis it that there is long-term trap response occurring in chub that have been caught repeatedly. Future analyses using data from arrays of PIT tag antennas spanning the width of the LCR may help test the second hypothesis.

While we find support for adult movement consistent with the residents and skip-spawning migrants present in theoretical models of partial migration, we also note low rates of movements between the LCR and Colorado River that suggest additional complexity. Thus, while some portions of the chub population are likely to show life histories similar to the extremes of life-long residency and rearing in the Colorado River and only returning to the LCR for spawning illustrated in Fig. 5, other portions of the population are likely to show intermediate growth rates (and survival). Likewise, while rates of movement between the LCR and the Colorado River for subadults and juveniles are generally low, they still suggest some movement. These low monthly rates compound over the course of a year and can lead to substantial annual fluxes of individuals. For example, when population sizes are in the thousands, a monthly movement rate around 0.01 could lead to a flux of hundreds of individuals over the course of a year.

The one exception to low monthly rates of movement prior to adulthood is the high probability of juveniles moving from the LCR to the Colorado River during the monsoon season. The effects of these movements on abundances of juveniles in the Colorado River study site are particularly clear in 2011 and 2012 when estimates of juvenile abundances increase by ∼4000 between July and September of each year (Appendix S3). The increase in abundances between July and September of 2010 (and between August and September 2009), in contrast, is much smaller, suggesting either that the export rate is not constant or that there was a smaller population of juveniles in the LCR to begin with in July. Interestingly, there was substantially more flooding during the monsoon season in 2011 and 2012 than in 2009 and 2010, suggesting a potential link between export and LCR flood frequency. Unfortunately, we are not able to estimate juvenile abundances in the LCR during July, and the relatively small number of marks put into the LCR during July does not allow us to estimate year-specific rates of outmigration over the study period. Ongoing research is increasing effort in July to obtain abundance estimates and allow for yearly estimates of outmigration rates.

An unresolved question in our river system regards the degree to which the resident versus the migrant populations drive overall dynamics of the partial migratory system. Based on our estimated rates, we expect that the two strategies would lead to similar reproductive results; however, we might also expect that the resident strategy would outperform the migrant strategy when the Colorado River is less hospitable (e.g., higher salmonid abundances or cooler water temperatures). As LCR residents would have a quicker generation time, they should also increase (or decrease) more quickly. On the other hand, 82% of the adult population (based on abundances reported in Appendix S3 and our estimate of *τ*) currently resides in the Colorado River during the nonspawning season, and vital rates under the environmental conditions experienced in 2009–2012 suggest that juveniles can successfully rear to adulthood in the Colorado River mainstem (Fig. 5). Survival was once thought to be uncommonly rare in the Colorado River because of the seasonally constant, low temperatures (Clarkson and Childs 2000).

One explanation is that under current environmental conditions, both strategies have roughly similar fitness's and are contributing to a fairly stable or slightly increasing adult population; however, when environmental conditions in the Colorado River decline, the migrating population in the Colorado River may become a population sink (sensu Pulliam 1988). Given the limited carrying capacity in the LCR (Meretsky et al. 2000; Benenati et al. 2002), this suggests that population targets in the system can only be met by managing the Colorado River to ensure relatively beneficial environmental conditions (e.g.*,* lower salmonid abundances or warmer water temperatures). Moreover, as system dynamics are relatively slow, conditions in the Colorado River will likely have to persist for long periods of time to change adult population sizes. Vital rates and abundances of smaller size classes will provide a leading indicator of future adult population size. However, given the long periods of time spent in these vulnerable life stages, a year or two of beneficial conditions can be easily negated by a change in these conditions. Future work will focus on exploring annual variation in vital rates, particularly as salmonid abundances in the Colorado River may be increasing.

## Supporting Information

**Appendix S1.** Annotated R code for fitting the global model described in the paper. **Appendix S2.** Tag retention study. **Appendix S3.** Abundance estimates. **Appendix S4.** GOF.
