# Supplementary material for: A quantitative life history of endangered humpback chub that spawn in the Little Colorado River: variation in movement, growth, and survival
Source: Ecol Evol. 2014 Feb 28;4(7):1006–18. doi: 10.1002/ece3.990 (PMC3997317; doi:10.1002/ece3.990)

Appendix A: Annotated R code for fitting the global model described in the paper

# the following function defines which parameters in the general model are being estimated independently.

ll_RJA<-function(par){

mu.g<-plogis(par[1:8]) # eight growth parameters

mu.s<-plogis(par[9:18]) # ten survival parameters

p<-plogis(par[19]) # tau in paper

mu.m<-plogis(par[20:33]) # 14 movement parameters (differs for other submodels)

beta<-plogis(par[34:175]) # capture probabilities

res.mu<- plogis(par[176:177]) # rates for potential residents (i.e., fish previously captured in LCR during fall)

g<-matrix(NA,nrow=8,ncol=42)

s<-matrix(NA,nrow=10,ncol=42)

m<-matrix(NA,nrow=12,ncol=42)

for (j in 1:8){g[j,]<-mu.g[j]} # state-specific, but time independent

for (j in 1:10){s[j,]<-mu.s[j]} # state-specific, but time independent

m[1,]<-mu.m[1]

m[1,c(4,5,16,17,28,29,40,41)]<-mu.m[2] # juvenile outmigration during monsoon season

m[2,]<-mu.m[3]

m[3,]<-mu.m[4]

m[4,]<-mu.m[5]

m[4,c(2,3,14,15,26,27,38,39)]<-mu.m[6] # emmigration rate after spawning season for size class 4 never seen in LCR during fall

m[5,]<-mu.m[7]

m[5,c(2,3,14,15,26,27,38,39)]<-mu.m[8] # emmigration rate after spawning season for size class 5 never seen in LCR during fall

m[6,]<-0

m[7,]<-mu.m[9]

m[8,]<-mu.m[10]

m[9,]<-mu.m[11]

m[9,c( 12, 24, 36)]<-mu.m[12] # spawning frequency (i.e. proportion of fish moving into LCR just before spawning) size class 4

m[10,]<-mu.m[13]

m[10,c( 12, 24, 36)]<-mu.m[14] # spawning frequency (i.e. proportion of fish moving into LCR just before spawning) size class 5

m[11,]<-res.mu[1] # movement rates out of LCR for potential residents - size class 4

m[12,]<-res.mu[2] # movement rates out of LCR for potential residents - size class 5

return(makepred(m=m,s=s,g=g,p=p,beta=beta))

}

# the following computes the likelihood

makepred<-function(g,s,m,p,beta){

phi<-array(NA,dim=c(16,16,42)) # monthly transition matrix – 16^th^ state is for dead individuals and is included for bookkeeping

phi.hv<-array(NA,dim=c(16,16,42)) # monthly transition matrix adjusted for monthly loss of vies in the LCR

phi.res<-array(0,dim=c(16,16,42)) # monthly transition matrix for adult fish previously caught in LCR (potential residents)

ot<-0.03 #ongoing monthly rate of tag loss for warm vie's

it<-0.03 #initial tag loss rate for vie and pit tags

iloss<-array(NA,dim=c(16,16,2)) # array to account for initial tag loss

iloss[,,1]<-diag(16) #old pit tags have a loss rate of 0

iloss[,,2]<-(1-it)*diag(16) # new tags have retention rate of (1-it)

iloss[,16,2]<-iloss[,16,2]+it

po<-array(NA,dim=c(16,11,22)) # matrix of capture probabilities

for (t in 1:42){

phi[1,1,t]<-s[1,t]*(1-g[1,t])*(1-m[1,t])

phi[1,2,t]<-s[1,t]*g[1,t]*(1-m[2,t])

phi[1,3:5,t]<-0

phi[1,6,t]<-s[1,t]*(1-g[1,t])*m[1,t]*p

phi[1,7,t]<-s[1,t]*g[1,t]*m[2,t]*p

phi[1,8:10,t]<-0

phi[1,11,t]<-s[1,t]*(1-g[1,t])*m[1,t]*(1-p)

phi[1,12,t]<-s[1,t]*g[1,t]*m[2,t]*(1-p)

phi[1,13:15,t]<-0

phi[1,16,t]<-1-s[1,t]

phi[2,1,t]<-0

phi[2,2,t]<-s[2,t]*(1-g[2,t])*(1-m[2,t])

phi[2,3,t]<-s[2,t]*g[2,t]*(1-m[3,t])

phi[2,4:6,t]<-0

phi[2,7,t]<-s[2,t]*(1-g[2,t])*m[2,t]*p

phi[2,8,t]<-s[2,t]*g[2,t]*m[3,t]*p

phi[2,9:11,t]<-0

phi[2,12,t]<-s[2,t]*(1-g[2,t])*m[2,t]*(1-p)

phi[2,13,t]<-s[2,t]*g[2,t]*m[3,t]*(1-p)

phi[2,14:15,t]<-0

phi[2,16,t]<-1-s[2,t]

phi[3,1:2,t]<-0

phi[3,3,t]<-s[3,t]*(1-g[3,t])*(1-m[3,t])

phi[3,4,t]<-s[3,t]*g[3,t]*(1-m[4,t])

phi[3,5:7,t]<-0

phi[3,8,t]<-s[3,t]*(1-g[3,t])*m[3,t]*p

phi[3,9,t]<-s[3,t]*g[3,t]*m[4,t]*p

phi[3,10:12,t]<-0

phi[3,13,t]<-s[3,t]*(1-g[3,t])*m[3,t]*(1-p)

phi[3,14,t]<-s[3,t]*g[3,t]*m[4,t]*(1-p)

phi[3,15,t]<-0

phi[3,16,t]<-1-s[3,t]

phi[4,1:3,t]<-0

phi[4,4,t]<-s[4,t]*(1-g[4,t])*(1-m[4,t])

phi[4,5,t]<-s[4,t]*g[4,t]*(1-m[5,t])

phi[4,6:8,t]<-0

phi[4,9,t]<-s[4,t]*(1-g[4,t])*m[4,t]*p

phi[4,10,t]<-s[4,t]*g[4,t]*m[5,t]*p

phi[4,11:13,t]<-0

phi[4,14,t]<-s[4,t]*(1-g[4,t])*m[4,t]*(1-p)

phi[4,15,t]<-s[4,t]*g[4,t]*m[5,t]*(1-p)

phi[4,16,t]<-1-s[4,t]

phi[5,1:4,t]<-0

phi[5,5,t]<-s[5,t]*(1-m[5,t])

phi[5,6:9,t]<-0

phi[5,10,t]<-s[5,t]*m[5,t]*p

phi[5,11:14,t]<-0

phi[5,15,t]<-s[5,t]*m[5,t]*(1-p)

phi[5,16,t]<-1-s[5,t]

phi[6,1,t]<-s[6,t]*(1-g[5,t])*m[6,t]

phi[6,2,t]<-s[6,t]*g[5,t]*m[7,t]

phi[6,3:5,t]<-0

phi[6,6,t]<-s[6,t]*(1-g[5,t])*(1-m[6,t])

phi[6,7,t]<-s[6,t]*g[5,t]*(1-m[7,t])

phi[6,8:15,t]<-0

phi[6,16,t]<-1-s[6,t]

phi[7,1,t]<-0

phi[7,2,t]<-s[7,t]*(1-g[6,t])*m[7,t]

phi[7,3,t]<-s[7,t]*g[6,t]*m[8,t]

phi[7,4:6,t]<-0

phi[7,7,t]<-s[7,t]*(1-g[6,t])*(1-m[7,t])

phi[7,8,t]<-s[7,t]*g[6,t]*(1-m[8,t])

phi[7,9:15,t]<-0

phi[7,16,t]<-1-s[7,t]

phi[8,1:2,t]<-0

phi[8,3,t]<-s[8,t]*(1-g[7,t])*m[8,t]

phi[8,4,t]<-s[8,t]*g[7,t]*m[9,t]

phi[8,5:7,t]<-0

phi[8,8,t]<-s[8,t]*(1-g[7,t])*(1-m[8,t])

phi[8,9,t]<-s[8,t]*g[7,t]*(1-m[9,t])

phi[8,10:15,t]<-0

phi[8,16,t]<-1-s[8,t]

phi[9,1:3,t]<-0

phi[9,4,t]<-s[9,t]*(1-g[8,t])*m[9,t]

phi[9,5,t]<-s[9,t]*g[8,t]*m[10,t]

phi[9,6:8,t]<-0

phi[9,9,t]<-s[9,t]*(1-g[8,t])*(1-m[9,t])

phi[9,10,t]<-s[9,t]*g[8,t]*(1-m[10,t])

phi[9,11:15,t]<-0

phi[9,16,t]<-1-s[9,t]

phi[10,1:4,t]<-0

phi[10,5,t]<-s[10,t]*m[10,t]

phi[10,6:9,t]<-0

phi[10,10,t]<-s[10,t]*(1-m[10,t])

phi[10,11:15,t]<-0

phi[10,16,t]<-1-s[10,t]

phi[11,1,t]<-s[6,t]*(1-g[5,t])*m[6,t]

phi[11,2,t]<-s[6,t]*g[5,t]*m[7,t]

phi[11,3:10,t]<-0

phi[11,11,t]<-s[6,t]*(1-g[5,t])*(1-m[6,t])

phi[11,12,t]<-s[6,t]*g[5,t]*(1-m[7,t])

phi[11,13:15,t]<-0

phi[11,16,t]<-1-s[6,t]

phi[12,1,t]<-0

phi[12,2,t]<-s[7,t]*(1-g[6,t])*m[7,t]

phi[12,3,t]<-s[7,t]*g[6,t]*m[8,t]

phi[12,4:11,t]<-0

phi[12,12,t]<-s[7,t]*(1-g[6,t])*(1-m[7,t])

phi[12,13,t]<-s[7,t]*g[6,t]*(1-m[8,t])

phi[12,14:15,t]<-0

phi[12,16,t]<-1-s[7,t]

phi[13,1:2,t]<-0

phi[13,3,t]<-s[8,t]*(1-g[7,t])*m[8,t]

phi[13,4,t]<-s[8,t]*g[7,t]*m[9,t]

phi[13,5:12,t]<-0

phi[13,13,t]<-s[8,t]*(1-g[7,t])*(1-m[8,t])

phi[13,14,t]<-s[8,t]*g[7,t]*(1-m[9,t])

phi[13,15,t]<-0

phi[13,16,t]<-1-s[8,t]

phi[14,1:3,t]<-0

phi[14,4,t]<-s[9,t]*(1-g[8,t])*m[9,t]

phi[14,5,t]<-s[9,t]*g[8,t]*m[10,t]

phi[14,6:13,t]<-0

phi[14,14,t]<-s[9,t]*(1-g[8,t])*(1-m[9,t])

phi[14,15,t]<-s[9,t]*g[8,t]*(1-m[10,t])

phi[14,16,t]<-1-s[9,t]

phi[15,1:4,t]<-0

phi[15,5,t]<-s[10,t]*m[10,t]

phi[15,6:14,t]<-0

phi[15,15,t]<-s[10,t]*(1-m[10,t])

phi[15,16,t]<-1-s[10,t]

for (j in 1:15){phi[16,j,t]<-0}

phi[16,16,t]<-1

phi.hv[1,1,t]<-s[1,t]*(1-g[1,t])*(1-m[1,t])*(1-ot)

phi.hv[1,2,t]<-s[1,t]*g[1,t]*(1-m[2,t])*(1-ot)

phi.hv[1,3:5,t]<-0

phi.hv[1,6,t]<-s[1,t]*(1-g[1,t])*m[1,t]*p*(1-ot)

phi.hv[1,7,t]<-s[1,t]*g[1,t]*m[2,t]*p*(1-ot)

phi.hv[1,8:10,t]<-0

phi.hv[1,11,t]<-s[1,t]*(1-g[1,t])*m[1,t]*(1-p)*(1-ot)

phi.hv[1,12,t]<-s[1,t]*g[1,t]*m[2,t]*(1-p)*(1-ot)

phi.hv[1,13:15,t]<-0

phi.hv[1,16,t]<-1-s[1,t]*(1-ot)

phi.hv[2,1,t]<-0

phi.hv[2,2,t]<-s[2,t]*(1-g[2,t])*(1-m[2,t])*(1-ot)

phi.hv[2,3,t]<-s[2,t]*g[2,t]*(1-m[3,t])*(1-ot)

phi.hv[2,4:6,t]<-0

phi.hv[2,7,t]<-s[2,t]*(1-g[2,t])*m[2,t]*p*(1-ot)

phi.hv[2,8,t]<-s[2,t]*g[2,t]*m[3,t]*p*(1-ot)

phi.hv[2,9:11,t]<-0

phi.hv[2,12,t]<-s[2,t]*(1-g[2,t])*m[2,t]*(1-p)*(1-ot)

phi.hv[2,13,t]<-s[2,t]*g[2,t]*m[3,t]*(1-p)*(1-ot)

phi.hv[2,14:15,t]<-0

phi.hv[2,16,t]<-1-s[2,t]*(1-ot)

phi.hv[3,1:2,t]<-0

phi.hv[3,3,t]<-s[3,t]*(1-g[3,t])*(1-m[3,t])*(1-ot)

phi.hv[3,4,t]<-s[3,t]*g[3,t]*(1-m[4,t])*(1-ot)

phi.hv[3,5:7,t]<-0

phi.hv[3,8,t]<-s[3,t]*(1-g[3,t])*m[3,t]*p*(1-ot)

phi.hv[3,9,t]<-s[3,t]*g[3,t]*m[4,t]*p*(1-ot)

phi.hv[3,10:12,t]<-0

phi.hv[3,13,t]<-s[3,t]*(1-g[3,t])*m[3,t]*(1-p)*(1-ot)

phi.hv[3,14,t]<-s[3,t]*g[3,t]*m[4,t]*(1-p)*(1-ot)

phi.hv[3,15,t]<-0

phi.hv[3,16,t]<-1-s[3,t]*(1-ot)

phi.hv[4,1:3,t]<-0

phi.hv[4,4,t]<-s[4,t]*(1-g[4,t])*(1-m[4,t])*(1-ot)

phi.hv[4,5,t]<-s[4,t]*g[4,t]*(1-m[5,t])*(1-ot)

phi.hv[4,6:8,t]<-0

phi.hv[4,9,t]<-s[4,t]*(1-g[4,t])*m[4,t]*p*(1-ot)

phi.hv[4,10,t]<-s[4,t]*g[4,t]*m[5,t]*p*(1-ot)

phi.hv[4,11:13,t]<-0

phi.hv[4,14,t]<-s[4,t]*(1-g[4,t])*m[4,t]*(1-p)*(1-ot)

phi.hv[4,15,t]<-s[4,t]*g[4,t]*m[5,t]*(1-p)*(1-ot)

phi.hv[4,16,t]<-1-s[4,t]*(1-ot)

phi.hv[5,1:4,t]<-0

phi.hv[5,5,t]<-s[5,t]*(1-m[5,t])*(1-ot)

phi.hv[5,6:9,t]<-0

phi.hv[5,10,t]<-s[5,t]*m[5,t]*p*(1-ot)

phi.hv[5,11:14,t]<-0

phi.hv[5,15,t]<-s[5,t]*m[5,t]*(1-p)*(1-ot)

phi.hv[5,16,t]<-1-s[5,t]*(1-ot)

phi.hv[6,1,t]<-s[6,t]*(1-g[5,t])*m[6,t]

phi.hv[6,2,t]<-s[6,t]*g[5,t]*m[7,t]

phi.hv[6,3:5,t]<-0

phi.hv[6,6,t]<-s[6,t]*(1-g[5,t])*(1-m[6,t])

phi.hv[6,7,t]<-s[6,t]*g[5,t]*(1-m[7,t])

phi.hv[6,8:15,t]<-0

phi.hv[6,16,t]<-1-s[6,t]

phi.hv[7,1,t]<-0

phi.hv[7,2,t]<-s[7,t]*(1-g[6,t])*m[7,t]

phi.hv[7,3,t]<-s[7,t]*g[6,t]*m[8,t]

phi.hv[7,4:6,t]<-0

phi.hv[7,7,t]<-s[7,t]*(1-g[6,t])*(1-m[7,t])

phi.hv[7,8,t]<-s[7,t]*g[6,t]*(1-m[8,t])

phi.hv[7,9:15,t]<-0

phi.hv[7,16,t]<-1-s[7,t]

phi.hv[8,1:2,t]<-0

phi.hv[8,3,t]<-s[8,t]*(1-g[7,t])*m[8,t]

phi.hv[8,4,t]<-s[8,t]*g[7,t]*m[9,t]

phi.hv[8,5:7,t]<-0

phi.hv[8,8,t]<-s[8,t]*(1-g[7,t])*(1-m[8,t])

phi.hv[8,9,t]<-s[8,t]*g[7,t]*(1-m[9,t])

phi.hv[8,10:15,t]<-0

phi.hv[8,16,t]<-1-s[8,t]

phi.hv[9,1:3,t]<-0

phi.hv[9,4,t]<-s[9,t]*(1-g[8,t])*m[9,t]

phi.hv[9,5,t]<-s[9,t]*g[8,t]*m[10,t]

phi.hv[9,6:8,t]<-0

phi.hv[9,9,t]<-s[9,t]*(1-g[8,t])*(1-m[9,t])

phi.hv[9,10,t]<-s[9,t]*g[8,t]*(1-m[10,t])

phi.hv[9,11:15,t]<-0

phi.hv[9,16,t]<-1-s[9,t]

phi.hv[10,1:4,t]<-0

phi.hv[10,5,t]<-s[10,t]*m[10,t]

phi.hv[10,6:9,t]<-0

phi.hv[10,10,t]<-s[10,t]*(1-m[10,t])

phi.hv[10,11:15,t]<-0

phi.hv[10,16,t]<-1-s[10,t]

phi.hv[11,1,t]<-s[6,t]*(1-g[5,t])*m[6,t]

phi.hv[11,2,t]<-s[6,t]*g[5,t]*m[7,t]

phi.hv[11,3:10,t]<-0

phi.hv[11,11,t]<-s[6,t]*(1-g[5,t])*(1-m[6,t])

phi.hv[11,12,t]<-s[6,t]*g[5,t]*(1-m[7,t])

phi.hv[11,13:15,t]<-0

phi.hv[11,16,t]<-1-s[6,t]

phi.hv[12,1,t]<-0

phi.hv[12,2,t]<-s[7,t]*(1-g[6,t])*m[7,t]

phi.hv[12,3,t]<-s[7,t]*g[6,t]*m[8,t]

phi.hv[12,4:11,t]<-0

phi.hv[12,12,t]<-s[7,t]*(1-g[6,t])*(1-m[7,t])

phi.hv[12,13,t]<-s[7,t]*g[6,t]*(1-m[8,t])

phi.hv[12,14:15,t]<-0

phi.hv[12,16,t]<-1-s[7,t]

phi.hv[13,1:2,t]<-0

phi.hv[13,3,t]<-s[8,t]*(1-g[7,t])*m[8,t]

phi.hv[13,4,t]<-s[8,t]*g[7,t]*m[9,t]

phi.hv[13,5:12,t]<-0

phi.hv[13,13,t]<-s[8,t]*(1-g[7,t])*(1-m[8,t])

phi.hv[13,14,t]<-s[8,t]*g[7,t]*(1-m[9,t])

phi.hv[13,15,t]<-0

phi.hv[13,16,t]<-1-s[8,t]

phi.hv[14,1:3,t]<-0

phi.hv[14,4,t]<-s[9,t]*(1-g[8,t])*m[9,t]

phi.hv[14,5,t]<-s[9,t]*g[8,t]*m[10,t]

phi.hv[14,6:13,t]<-0

phi.hv[14,14,t]<-s[9,t]*(1-g[8,t])*(1-m[9,t])

phi.hv[14,15,t]<-s[9,t]*g[8,t]*(1-m[10,t])

phi.hv[14,16,t]<-1-s[9,t]

phi.hv[15,1:4,t]<-0

phi.hv[15,5,t]<-s[10,t]*m[10,t]

phi.hv[15,6:14,t]<-0

phi.hv[15,15,t]<-s[10,t]*(1-m[10,t])

phi.hv[15,16,t]<-1-s[10,t]

for (j in 1:15){phi.hv[16,j,t]<-0}

phi.hv[16,16,t]<-1

phi.res[4,1:3,t]<-0

phi.res[4,4,t]<-s[4,t]*(1-g[4,t])*(1-m[11,t])

phi.res[4,5,t]<-s[4,t]*g[4,t]*(1-m[12,t])

phi.res[4,6:8,t]<-0

phi.res[4,9,t]<-s[4,t]*(1-g[4,t])*m[11,t]*p

phi.res[4,10,t]<-s[4,t]*g[4,t]*m[12,t]*p

phi.res[4,11:13,t]<-0

phi.res[4,14,t]<-s[4,t]*(1-g[4,t])*m[11,t]*(1-p)

phi.res[4,15,t]<-s[4,t]*g[4,t]*m[12,t]*(1-p)

phi.res[4,16,t]<-1-s[4,t]

phi.res[5,1:4,t]<-0

phi.res[5,5,t]<-s[5,t]*(1-m[12,t])

phi.res[5,6:9,t]<-0

phi.res[5,10,t]<-s[5,t]*m[12,t]*p

phi.res[5,11:14,t]<-0

phi.res[5,15,t]<-s[5,t]*m[12,t]*(1-p)

phi.res[5,16,t]<-1-s[5,t]

phi.res[9,1:3,t]<-0

phi.res[9,4,t]<-s[9,t]*(1-g[8,t])*m[9,t]

phi.res[9,5,t]<-s[9,t]*g[8,t]*m[10,t]

phi.res[9,6:8,t]<-0

phi.res[9,9,t]<-s[9,t]*(1-g[8,t])*(1-m[9,t])

phi.res[9,10,t]<-s[9,t]*g[8,t]*(1-m[10,t])

phi.res[9,11:15,t]<-0

phi.res[9,16,t]<-1-s[9,t]

phi.res[10,1:4,t]<-0

phi.res[10,5,t]<-s[10,t]*m[10,t]

phi.res[10,6:9,t]<-0

phi.res[10,10,t]<-s[10,t]*(1-m[10,t])

phi.res[10,11:15,t]<-0

phi.res[10,16,t]<-1-s[10,t]

phi.res[14,1:3,t]<-0

phi.res[14,4,t]<-s[9,t]*(1-g[8,t])*m[9,t]

phi.res[14,5,t]<-s[9,t]*g[8,t]*m[10,t]

phi.res[14,6:13,t]<-0

phi.res[14,14,t]<-s[9,t]*(1-g[8,t])*(1-m[9,t])

phi.res[14,15,t]<-s[9,t]*g[8,t]*(1-m[10,t])

phi.res[14,16,t]<-1-s[9,t]

phi.res[15,1:4,t]<-0

phi.res[15,5,t]<-s[10,t]*m[10,t]

phi.res[15,6:14,t]<-0

phi.res[15,15,t]<-s[10,t]*(1-m[10,t])

phi.res[15,16,t]<-1-s[10,t]

for (j in 1:15){phi.res[16,j,t]<-0}

phi.res[16,16,t]<-1

}

# below code aggregates monthly transition matrices during which no observations were made to ease computations

temp<-array(NA,dim=c(16,16,9))

temp2<-array(NA,dim=c(16,16,3))

temp[,,1]<-phi[,,7]%*%phi[,,8]

temp[,,2]<-phi[,,9]%*%phi[,,10]

temp[,,3]<-phi[,,11]%*%phi[,,12]

temp[,,4]<-phi[,,19]%*%phi[,,20]

temp[,,5]<-phi[,,21]%*%phi[,,22]

temp[,,6]<-phi[,,23]%*%phi[,,24]

temp[,,7]<-phi[,,31]%*%phi[,,32]

temp[,,8]<-phi[,,33]%*%phi[,,34]

temp[,,9]<-phi[,,35]%*%phi[,,36]

temp2[,,1]<-temp[,,1]%*%temp[,,2]

temp2[,,2]<-temp[,,4]%*%temp[,,5]

temp2[,,3]<-temp[,,7]%*%temp[,,8]

ophi<-array(NA,dim=c(16,16,22,3)) # last dimension is for not hv or hv or res

ophi[,,1,1]<-phi[,,1] #A-M

ophi[,,2,1]<-phi[,,2]%*%phi[,,3] #M-J-J

ophi[,,3,1]<-phi[,,4] #J-A

ophi[,,4,1]<-phi[,,5] #A-S

ophi[,,5,1]<-phi[,,6]#S-O

ophi[,,6,1]<-temp2[,,1]%*%temp[,,3] #O-A

ophi[,,7,1]<-phi[,,13] #A-M

ophi[,,8,1]<-phi[,,14]%*%phi[,,15]

ophi[,,9,1]<-phi[,,16] #J-A

ophi[,,10,1]<-phi[,,17] #A-S

ophi[,,11,1]<-phi[,,18] #S-O

ophi[,,12,1]<-temp2[,,2]%*%temp[,,6] #O-A

ophi[,,13,1]<-phi[,,25] #A-M

ophi[,,14,1]<-phi[,,26]%*%phi[,,27]

ophi[,,15,1]<-phi[,,28] #J-A

ophi[,,16,1]<-phi[,,29] #A-S

ophi[,,17,1]<-phi[,,30] #S-O

ophi[,,18,1]<-temp2[,,3]%*%temp[,,9] #O-A

ophi[,,19,1]<-phi[,,37] #A-M

ophi[,,20,1]<-phi[,,38]%*%phi[,,39] #M-J-J

ophi[,,21,1]<-phi[,,40]%*%phi[,,41] #J-A-S

ophi[,,22,1]<-phi[,,42] #S-O

temp.hv<-array(NA,dim=c(16,16,9))

temp2.hv<-array(NA,dim=c(16,16,3))

temp.hv[,,1]<-phi.hv[,,7]%*%phi.hv[,,8]

temp.hv[,,2]<-phi.hv[,,9]%*%phi.hv[,,10]

temp.hv[,,3]<-phi.hv[,,11]%*%phi.hv[,,12]

temp.hv[,,4]<-phi.hv[,,19]%*%phi.hv[,,20]

temp.hv[,,5]<-phi.hv[,,21]%*%phi.hv[,,22]

temp.hv[,,6]<-phi.hv[,,23]%*%phi.hv[,,24]

temp.hv[,,7]<-phi.hv[,,31]%*%phi.hv[,,32]

temp.hv[,,8]<-phi.hv[,,33]%*%phi.hv[,,34]

temp.hv[,,9]<-phi.hv[,,35]%*%phi.hv[,,36]

temp2.hv[,,1]<-temp.hv[,,1]%*%temp.hv[,,2]

temp2.hv[,,2]<-temp.hv[,,4]%*%temp.hv[,,5]

temp2.hv[,,3]<-temp.hv[,,7]%*%temp.hv[,,8]

ophi[,,1,2]<-phi.hv[,,1] #A-M

ophi[,,2,2]<-phi.hv[,,2]%*%phi.hv[,,3] #M-J-J

ophi[,,3,2]<-phi.hv[,,4] #J-A

ophi[,,4,2]<-phi.hv[,,5] #A-S

ophi[,,5,2]<-phi.hv[,,6]#S-O

ophi[,,6,2]<-temp2.hv[,,1]%*%temp.hv[,,3] #O-A

ophi[,,7,2]<-phi.hv[,,13] #A-M

ophi[,,8,2]<-phi.hv[,,14]%*%phi.hv[,,15]

ophi[,,9,2]<-phi.hv[,,16] #J-A

ophi[,,10,2]<-phi.hv[,,17] #A-S

ophi[,,11,2]<-phi.hv[,,18] #S-O

ophi[,,12,2]<-temp2.hv[,,2]%*%temp.hv[,,6] #O-A

ophi[,,13,2]<-phi.hv[,,25] #A-M

ophi[,,14,2]<-phi.hv[,,26]%*%phi.hv[,,27]

ophi[,,15,2]<-phi.hv[,,28] #J-A

ophi[,,16,2]<-phi.hv[,,29] #A-S

ophi[,,17,2]<-phi.hv[,,30] #S-O

ophi[,,18,2]<-temp2.hv[,,3]%*%temp.hv[,,9] #O-A

ophi[,,19,2]<-phi.hv[,,37] #A-M

ophi[,,20,2]<-phi.hv[,,38]%*%phi.hv[,,39] #M-J-J

ophi[,,21,2]<-phi.hv[,,40]%*%phi.hv[,,41] #J-A-S

ophi[,,22,2]<-phi.hv[,,42] #S-O

#

temp.res<-array(NA,dim=c(16,16,9))

temp2.res<-array(NA,dim=c(16,16,3))

temp.res[,,1]<-phi.res[,,7]%*%phi.res[,,8]

temp.res[,,2]<-phi.res[,,9]%*%phi.res[,,10]

temp.res[,,3]<-phi.res[,,11]%*%phi.res[,,12]

temp.res[,,4]<-phi.res[,,19]%*%phi.res[,,20]

temp.res[,,5]<-phi.res[,,21]%*%phi.res[,,22]

temp.res[,,6]<-phi.res[,,23]%*%phi.res[,,24]

temp.res[,,7]<-phi.res[,,31]%*%phi.res[,,32]

temp.res[,,8]<-phi.res[,,33]%*%phi.res[,,34]

temp.res[,,9]<-phi.res[,,35]%*%phi.res[,,36]

temp2.res[,,1]<-temp.res[,,1]%*%temp.res[,,2]

temp2.res[,,2]<-temp.res[,,4]%*%temp.res[,,5]

temp2.res[,,3]<-temp.res[,,7]%*%temp.res[,,8]

ophi[,,1,3]<-phi.res[,,1] #A-M

ophi[,,2,3]<-phi.res[,,2]%*%phi.res[,,3] #M-J-J

ophi[,,3,3]<-phi.res[,,4] #J-A

ophi[,,4,3]<-phi.res[,,5] #A-S

ophi[,,5,3]<-phi.res[,,6]#S-O

ophi[,,6,3]<-temp2.res[,,1]%*%temp.res[,,3] #O-A

ophi[,,7,3]<-phi.res[,,13] #A-M

ophi[,,8,3]<-phi.res[,,14]%*%phi.res[,,15]

ophi[,,9,3]<-phi.res[,,16] #J-A

ophi[,,10,3]<-phi.res[,,17] #A-S

ophi[,,11,3]<-phi.res[,,18] #S-O

ophi[,,12,3]<-temp2.res[,,2]%*%temp.res[,,6] #O-A

ophi[,,13,3]<-phi.res[,,25] #A-M

ophi[,,14,3]<-phi.res[,,26]%*%phi.res[,,27]

ophi[,,15,3]<-phi.res[,,28] #J-A

ophi[,,16,3]<-phi.res[,,29] #A-S

ophi[,,17,3]<-phi.res[,,30] #S-O

ophi[,,18,3]<-temp2.res[,,3]%*%temp.res[,,9] #O-A

ophi[,,19,3]<-phi.res[,,37] #A-M

ophi[,,20,3]<-phi.res[,,38]%*%phi.res[,,39] #M-J-J

ophi[,,21,3]<-phi.res[,,40]%*%phi.res[,,41] #J-A-S

ophi[,,22,3]<-phi.res[,,42] #S-O

# below defines which states and time periods are represented by each capture probability beta

for (j in 11:16){

for (t in 1:22){

po[j,11,t]<-1

po[j,1:10,t]<-0}}

for (j in 1:9){

for (k in (j+1):10){

for (t in 1:22){po[j,k,t]<-0}}}

for (j in 2:10){

for (k in 1:(j-1)){

for (t in 1:22){po[j,k,t]<-0}}}

po[1,1,1]<-0

po[1,1,2]<-0

po[1,1,3]<-0

po[1,1,4]<-beta[1]

po[1,1,5]<-beta[2]

po[1,1,6]<-0

po[1,1,7]<-0

po[1,1,8]<-0

po[1,1,9]<-0

po[1,1,10]<-beta[3]

po[1,1,11]<-beta[4]

po[1,1,12]<-0

po[1,1,13]<-0

po[1,1,14]<-0

po[1,1,15]<-0

po[1,1,16]<-beta[5]

po[1,1,17]<-beta[6]

po[1,1,18]<-0

po[1,1,19]<-0

po[1,1,20]<-0

po[1,1,21]<-beta[7]

po[1,1,22]<-beta[8]

po[2,2,1]<-beta[9]

po[2,2,2]<-0

po[2,2,3]<-0

po[2,2,4]<-beta[10]

po[2,2,5]<-beta[11]

po[2,2,6]<-beta[12]

po[2,2,7]<-beta[13]

po[2,2,8]<-0

po[2,2,9]<-0

po[2,2,10]<-beta[14]

po[2,2,11]<-beta[15]

po[2,2,12]<-beta[16]

po[2,2,13]<-beta[17]

po[2,2,14]<-0

po[2,2,15]<-0

po[2,2,16]<-beta[18]

po[2,2,17]<-beta[19]

po[2,2,18]<-beta[20]

po[2,2,19]<-beta[21]

po[2,2,20]<-0

po[2,2,21]<-beta[22]

po[2,2,22]<-beta[23]

po[3,3,1]<-beta[24]

po[3,3,2]<-0

po[3,3,3]<-0

po[3,3,4]<-beta[25]

po[3,3,5]<-beta[26]

po[3,3,6]<-beta[27]

po[3,3,7]<-beta[28]

po[3,3,8]<-0

po[3,3,9]<-0

po[3,3,10]<-beta[29]

po[3,3,11]<-beta[30]

po[3,3,12]<-beta[31]

po[3,3,13]<-beta[32]

po[3,3,14]<-0

po[3,3,15]<-0

po[3,3,16]<-beta[33]

po[3,3,17]<-beta[34]

po[3,3,18]<-beta[35]

po[3,3,19]<-beta[36]

po[3,3,20]<-0

po[3,3,21]<-beta[37]

po[3,3,22]<-beta[38]

po[4,4,1]<-beta[39]

po[4,4,2]<-0

po[4,4,3]<-0

po[4,4,4]<-beta[40]

po[4,4,5]<-beta[41]

po[4,4,6]<-beta[42]

po[4,4,7]<-beta[43]

po[4,4,8]<-0

po[4,4,9]<-0

po[4,4,10]<-beta[44]

po[4,4,11]<-beta[45]

po[4,4,12]<-beta[46]

po[4,4,13]<-beta[47]

po[4,4,14]<-0

po[4,4,15]<-0

po[4,4,16]<-beta[48]

po[4,4,17]<-beta[49]

po[4,4,18]<-beta[50]

po[4,4,19]<-beta[51]

po[4,4,20]<-0

po[4,4,21]<-beta[52]

po[4,4,22]<-beta[53]

po[5,5,1]<-beta[54]

po[5,5,2]<-0

po[5,5,3]<-0

po[5,5,4]<-beta[55]

po[5,5,5]<-beta[56]

po[5,5,6]<-beta[57]

po[5,5,7]<-beta[58]

po[5,5,8]<-0

po[5,5,9]<-0

po[5,5,10]<-beta[59]

po[5,5,11]<-beta[60]

po[5,5,12]<-beta[61]

po[5,5,13]<-beta[62]

po[5,5,14]<-0

po[5,5,15]<-0

po[5,5,16]<-beta[63]

po[5,5,17]<-beta[64]

po[5,5,18]<-beta[65]

po[5,5,19]<-beta[66]

po[5,5,20]<-0

po[5,5,21]<-beta[67]

po[5,5,22]<-beta[68]

po[6,6,1]<-0

po[6,6,2]<-0

po[6,6,3]<-beta[69]

po[6,6,4]<-beta[70]

po[6,6,5]<-beta[71]

po[6,6,6]<-0

po[6,6,7]<-0

po[6,6,8]<-beta[72]

po[6,6,9]<-beta[73]

po[6,6,10]<-beta[74]

po[6,6,11]<-beta[75]

po[6,6,12]<-0

po[6,6,13]<-0

po[6,6,14]<-beta[76]

po[6,6,15]<-beta[77]

po[6,6,16]<-beta[78]

po[6,6,17]<-beta[79]

po[6,6,18]<-beta[80]

po[6,6,19]<-0

po[6,6,20]<-beta[81]

po[6,6,21]<-beta[82]

po[6,6,22]<-0

po[7,7,1]<-0

po[7,7,2]<-beta[83]

po[7,7,3]<-beta[84]

po[7,7,4]<-beta[85]

po[7,7,5]<-beta[86]

po[7,7,6]<-0

po[7,7,7]<-0

po[7,7,8]<-beta[87]

po[7,7,9]<-beta[88]

po[7,7,10]<-beta[89]

po[7,7,11]<-beta[90]

po[7,7,12]<-0

po[7,7,13]<-0

po[7,7,14]<-beta[91]

po[7,7,15]<-beta[92]

po[7,7,16]<-beta[93]

po[7,7,17]<-beta[94]

po[7,7,18]<-beta[95]

po[7,7,19]<-0

po[7,7,20]<-beta[96]

po[7,7,21]<-beta[97]

po[7,7,22]<-0

po[8,8,1]<-0

po[8,8,2]<-beta[98]

po[8,8,3]<-beta[99]

po[8,8,4]<-beta[100]

po[8,8,5]<-beta[101]

po[8,8,6]<-0

po[8,8,7]<-0

po[8,8,8]<-beta[102]

po[8,8,9]<-beta[103]

po[8,8,10]<-beta[104]

po[8,8,11]<-beta[105]

po[8,8,12]<-0

po[8,8,13]<-0

po[8,8,14]<-beta[106]

po[8,8,15]<-beta[107]

po[8,8,16]<-beta[108]

po[8,8,17]<-beta[109]

po[8,8,18]<-beta[110]

po[8,8,19]<-0

po[8,8,20]<-beta[111]

po[8,8,21]<-beta[112]

po[8,8,22]<-0

po[9,9,1]<-0

po[9,9,2]<-beta[113]

po[9,9,3]<-beta[114]

po[9,9,4]<-beta[115]

po[9,9,5]<-beta[116]

po[9,9,6]<-0

po[9,9,7]<-0

po[9,9,8]<-beta[117]

po[9,9,9]<-beta[118]

po[9,9,10]<-beta[119]

po[9,9,11]<-beta[120]

po[9,9,12]<-0

po[9,9,13]<-0

po[9,9,14]<-beta[121]

po[9,9,15]<-beta[122]

po[9,9,16]<-beta[123]

po[9,9,17]<-beta[124]

po[9,9,18]<-beta[125]

po[9,9,19]<-0

po[9,9,20]<-beta[126]

po[9,9,21]<-beta[127]

po[9,9,22]<-0

po[10,10,1]<-0

po[10,10,2]<-beta[128]

po[10,10,3]<-beta[129]

po[10,10,4]<-beta[130]

po[10,10,5]<-beta[131]

po[10,10,6]<-0

po[10,10,7]<-0

po[10,10,8]<-beta[132]

po[10,10,9]<-beta[133]

po[10,10,10]<-beta[134]

po[10,10,11]<-beta[135]

po[10,10,12]<-0

po[10,10,13]<-0

po[10,10,14]<-beta[136]

po[10,10,15]<-beta[137]

po[10,10,16]<-beta[138]

po[10,10,17]<-beta[139]

po[10,10,18]<-beta[140]

po[10,10,19]<-0

po[10,10,20]<-beta[141]

po[10,10,21]<-beta[142]

po[10,10,22]<-0

for (j in 1:10){

for (t in 1:22){

po[j,11,t]<-1-po[j,j,t]

}}

#below keeps track of probability of being in a state and not observed

trophi<-array(NA,dim=c(16,16,21,21,3)) ##last dimension for not hv versus hv

for (k in 1:21){

for (i in 1:16){

for (j in 1:16){

trophi[i,j,k,1,1]<-ophi[i,j,k,1]*po[j,11,k]}}}

for (k in 1:20){trophi[,,k,2,1]<-trophi[,,k,1,1]%*%trophi[,,(k+1),1,1]}

for (k in 1:19){trophi[,,k,3,1]<-trophi[,,k,2,1]%*%trophi[,,(k+2),1,1]}

for (k in 1:18){trophi[,,k,4,1]<-trophi[,,k,3,1]%*%trophi[,,(k+3),1,1]}

for (k in 1:17){trophi[,,k,5,1]<-trophi[,,k,4,1]%*%trophi[,,(k+4),1,1]}

for (k in 1:16){trophi[,,k,6,1]<-trophi[,,k,5,1]%*%trophi[,,(k+5),1,1]}

for (k in 1:15){trophi[,,k,7,1]<-trophi[,,k,6,1]%*%trophi[,,(k+6),1,1]}

for (k in 1:14){trophi[,,k,8,1]<-trophi[,,k,7,1]%*%trophi[,,(k+7),1,1]}

for (k in 1:13){trophi[,,k,9,1]<-trophi[,,k,8,1]%*%trophi[,,(k+8),1,1]}

for (k in 1:12){trophi[,,k,10,1]<-trophi[,,k,9,1]%*%trophi[,,(k+9),1,1]}

for (k in 1:11){trophi[,,k,11,1]<-trophi[,,k,10,1]%*%trophi[,,(k+10),1,1]}

for (k in 1:10){trophi[,,k,12,1]<-trophi[,,k,11,1]%*%trophi[,,(k+11),1,1]}

for (k in 1:9){trophi[,,k,13,1]<-trophi[,,k,12,1]%*%trophi[,,(k+12),1,1]}

for (k in 1:8){trophi[,,k,14,1]<-trophi[,,k,13,1]%*%trophi[,,(k+13),1,1]}

for (k in 1:7){trophi[,,k,15,1]<-trophi[,,k,14,1]%*%trophi[,,(k+14),1,1]}

for (k in 1:6){trophi[,,k,16,1]<-trophi[,,k,15,1]%*%trophi[,,(k+15),1,1]}

for (k in 1:5){trophi[,,k,17,1]<-trophi[,,k,16,1]%*%trophi[,,(k+16),1,1]}

for (k in 1:4){trophi[,,k,18,1]<-trophi[,,k,17,1]%*%trophi[,,(k+17),1,1]}

for (k in 1:3){trophi[,,k,19,1]<-trophi[,,k,18,1]%*%trophi[,,(k+18),1,1]}

for (k in 1:2){trophi[,,k,20,1]<-trophi[,,k,19,1]%*%trophi[,,(k+19),1,1]}

trophi[,,1,21,1]<-trophi[,,1,20,1]%*%trophi[,,21,1,1]

for (k in 1:21){

for (i in 1:16){

for (j in 1:16){

trophi[i,j,k,1,2]<-ophi[i,j,k,2]*po[j,11,k]}}}

for (k in 1:20){trophi[,,k,2,2]<-trophi[,,k,1,2]%*%trophi[,,(k+1),1,2]}

for (k in 1:19){trophi[,,k,3,2]<-trophi[,,k,2,2]%*%trophi[,,(k+2),1,2]}

for (k in 1:18){trophi[,,k,4,2]<-trophi[,,k,3,2]%*%trophi[,,(k+3),1,2]}

for (k in 1:17){trophi[,,k,5,2]<-trophi[,,k,4,2]%*%trophi[,,(k+4),1,2]}

for (k in 1:16){trophi[,,k,6,2]<-trophi[,,k,5,2]%*%trophi[,,(k+5),1,2]}

for (k in 1:15){trophi[,,k,7,2]<-trophi[,,k,6,2]%*%trophi[,,(k+6),1,2]}

for (k in 1:14){trophi[,,k,8,2]<-trophi[,,k,7,2]%*%trophi[,,(k+7),1,2]}

for (k in 1:13){trophi[,,k,9,2]<-trophi[,,k,8,2]%*%trophi[,,(k+8),1,2]}

for (k in 1:12){trophi[,,k,10,2]<-trophi[,,k,9,2]%*%trophi[,,(k+9),1,2]}

for (k in 1:11){trophi[,,k,11,2]<-trophi[,,k,10,2]%*%trophi[,,(k+10),1,2]}

for (k in 1:10){trophi[,,k,12,2]<-trophi[,,k,11,2]%*%trophi[,,(k+11),1,2]}

for (k in 1:9){trophi[,,k,13,2]<-trophi[,,k,12,2]%*%trophi[,,(k+12),1,2]}

for (k in 1:8){trophi[,,k,14,2]<-trophi[,,k,13,2]%*%trophi[,,(k+13),1,2]}

for (k in 1:7){trophi[,,k,15,2]<-trophi[,,k,14,2]%*%trophi[,,(k+14),1,2]}

for (k in 1:6){trophi[,,k,16,2]<-trophi[,,k,15,2]%*%trophi[,,(k+15),1,2]}

for (k in 1:5){trophi[,,k,17,2]<-trophi[,,k,16,2]%*%trophi[,,(k+16),1,2]}

for (k in 1:4){trophi[,,k,18,2]<-trophi[,,k,17,2]%*%trophi[,,(k+17),1,2]}

for (k in 1:3){trophi[,,k,19,2]<-trophi[,,k,18,2]%*%trophi[,,(k+18),1,2]}

for (k in 1:2){trophi[,,k,20,2]<-trophi[,,k,19,2]%*%trophi[,,(k+19),1,2]}

trophi[,,1,21,2]<-trophi[,,1,20,2]%*%trophi[,,21,1,2]

for (k in 1:21){

for (i in 1:16){

for (j in 1:16){

trophi[i,j,k,1,3]<-ophi[i,j,k,3]*po[j,11,k]}}}

for (k in 1:20){trophi[,,k,2,3]<-trophi[,,k,1,3]%*%trophi[,,(k+1),1,3]}

for (k in 1:19){trophi[,,k,3,3]<-trophi[,,k,2,3]%*%trophi[,,(k+2),1,3]}

for (k in 1:18){trophi[,,k,4,3]<-trophi[,,k,3,3]%*%trophi[,,(k+3),1,3]}

for (k in 1:17){trophi[,,k,5,3]<-trophi[,,k,4,3]%*%trophi[,,(k+4),1,3]}

for (k in 1:16){trophi[,,k,6,3]<-trophi[,,k,5,3]%*%trophi[,,(k+5),1,3]}

for (k in 1:15){trophi[,,k,7,3]<-trophi[,,k,6,3]%*%trophi[,,(k+6),1,3]}

for (k in 1:14){trophi[,,k,8,3]<-trophi[,,k,7,3]%*%trophi[,,(k+7),1,3]}

for (k in 1:13){trophi[,,k,9,3]<-trophi[,,k,8,3]%*%trophi[,,(k+8),1,3]}

for (k in 1:12){trophi[,,k,10,3]<-trophi[,,k,9,3]%*%trophi[,,(k+9),1,3]}

for (k in 1:11){trophi[,,k,11,3]<-trophi[,,k,10,3]%*%trophi[,,(k+10),1,3]}

for (k in 1:10){trophi[,,k,12,3]<-trophi[,,k,11,3]%*%trophi[,,(k+11),1,3]}

for (k in 1:9){trophi[,,k,13,3]<-trophi[,,k,12,3]%*%trophi[,,(k+12),1,3]}

for (k in 1:8){trophi[,,k,14,3]<-trophi[,,k,13,3]%*%trophi[,,(k+13),1,3]}

for (k in 1:7){trophi[,,k,15,3]<-trophi[,,k,14,3]%*%trophi[,,(k+14),1,3]}

for (k in 1:6){trophi[,,k,16,3]<-trophi[,,k,15,3]%*%trophi[,,(k+15),1,3]}

for (k in 1:5){trophi[,,k,17,3]<-trophi[,,k,16,3]%*%trophi[,,(k+16),1,3]}

for (k in 1:4){trophi[,,k,18,3]<-trophi[,,k,17,3]%*%trophi[,,(k+17),1,3]}

for (k in 1:3){trophi[,,k,19,3]<-trophi[,,k,18,3]%*%trophi[,,(k+18),1,3]}

for (k in 1:2){trophi[,,k,20,3]<-trophi[,,k,19,3]%*%trophi[,,(k+19),1,3]}

trophi[,,1,21,3]<-trophi[,,1,20,3]%*%trophi[,,21,1,3]

llik<-numeric()

#following function determines log-likelihood based on observed capture histories (CH), depending on whether fish was marked with vie in LCR (hV) or previously observed in the LCR during fall (pot.res). Capture histories included 10 observed states and 11 when an individual was not observed in that time period. vector new tag has zeros for old tags and 1 for new tags

ifcn<-function(k){

start<-which(CH[k,]!=11)[1]

end<-ifelse(length(which(CH[k,]!=11))==1,23,which(CH[k,]!=11)[2])

dif<-end-start-1

if (dif==0&pot.res[k]==0) {pred<-log((iloss[,,(newtag[k]+1)]%*%ophi[,,start,(1+hV[k])])[CH[k,start],]%*%po[,CH[k,(start+1)],start])}

else {

if (pot.res[k]==0) {tpred<-(iloss[,,(newtag[k]+1)]%*%trophi[,,start,dif,(1+hV[k])])[CH[k,start],]%*%ophi[,,(start+dif),(1+hV[k])]

pred<-log(tpred%*%po[,CH[k,(start+dif+1)],(start+dif)])}

else {

if (dif==0&pot.res[k]==1) {pred<-log((iloss[,,(newtag[k]+1)]%*%ophi[,,start,3])[CH[k,start],]%*%po[,CH[k,(start+1)],start])}

else {tpred<-(iloss[,,(newtag[k]+1)]%*%trophi[,,start,dif,3])[CH[k,start],]%*%ophi[,,(start+dif),3]

pred<-log(tpred%*%po[,CH[k,(start+dif+1)],(start+dif)])}}

}

return(pred)}

for (j in 1:NCH){llik[j]<-ifcn(j)} # log-likelihood for each observed capture history (NCH – number of unique capture histories)

return(sum(-1*llik*(FR)))

}

Appendix B: Tag retention study

300 Roundtail chub (*Gila robusta*) were given VIE marks and then split into two treatments (warm and cold water) and stored in tanks with 100 unmarked roundtail chub (50 per treatment). Tanks were checked two days after tagging to determine initial mortality and tag loss and 98 days after tagging to determine long-term retention rates. Since marked and unmarked fish were stored together determining retention rates is not straightforward and was calculated using a model with up to eight parameters: initial tagging mortality ($\iota_{R}$), initial tag loss ($\kappa_{R}$), delayed daily tag loss ($\nu_{R}$) and a daily survival rate in the absence of tagging mortality ($\varsigma_{R}$), which in the most general version of the model were estimated independently for each treatment, R. Data were the number of marked ($M_{R,t}$) and unmarked ($U_{R,t}$) fish in each treatment, R, at t = 2 and t = 98 days. Predictions for these data based on model parameters are as follows:

$$M_{R,2}=M_{R,0}\left( 1-\iota_{R} \right)(1-\kappa_{R}){\varsigma_{R}}^{2}$$

$$U_{R,2}=(M_{R,0}\left( 1-\iota_{R} \right)\kappa_{R}+U_{R,0}){\varsigma_{R}}^{2}$$

$$M_{R,98}=M_{R,2}*{\varsigma_{R}}^{98}\left( 1-\nu_{R} \right)^{98}$$

$$U_{R,98}=(M_{R,2}(1-\left( 1-\nu_{R} \right)^{98})+U_{R,2}){\varsigma_{R}}^{98}$$

We developed five candidate models including: 1) a global model in which all eight parameters were estimated; 2) a model in which $\iota_{R}$ and $\kappa_{R}$ did not vary between treatments, but $\varsigma_{R}$ and $\nu_{R}$ did vary; 3) a model in which all four parameters did not vary between treatments, 4) a model in which $\iota_{R}$, $\kappa_{R}$ and $\varsigma_{R}$ did not vary between treatments and $\nu_{R}$was set equal to zero for the cold water treatment, 5) a model in which $\iota_{R}$, $\kappa_{R}$ and $\varsigma_{R}$ did not vary between treatments and $\nu_{R}$ was set equal to zero for both treatments. Model values were fit via maximum likelihood assuming a Poisson error distribution and model selection was based on AIC. We used the sum of ι and κ as an estimate of δ and scaled ν, a daily rate, to π, the monthly (30-day) rate for the multistate model. The best model was the fourth model, and the next best models were models three and five, both of which were separated from model four by 3 AIC. We present the data, and parameter estimates from the model, as well as the value of the derived parameters δ and π below.

| Treatment | $M_{R,2}$ | $U_{R,2}$ | $M_{R,98}$ | $U_{R,98}$ | $\iota_{R}$ | $\kappa_{R}$ | $\varsigma_{R}$ | $\nu_{R}$ | δ | π |
| --- | --- | --- | --- | --- | --- | --- | --- | --- | --- | --- |
| Cold | 145 | 52 | 140 | 48 | 0.0225 | 0.0070 | 0.9997 | 0.00105 | 0.0295 | 0.0310 |
| Warm | 146 | 50 | 129 | 63 | 0.0225 | 0.0070 | 0.9997 | 0 (not estimated) | 0.0295 | 0 |

Appendix C: Abundance estimates

*Methods:* We calculated state-specific abundances for each period where data were available by dividing catch (recaptures and captures combined) in that state by the estimated capture probability for that state and time period. We calculated the total adult abundance for September of each year (only month in which estimates of LCR and Colorado River HBC monitoring site abundances were both available in all 4 years) using the following equation:

$$\hat{N_{t}^{adult}}=\frac{C_{t}^{4}}{\hat{p_{t}^{4}}}+\frac{C_{t}^{5}}{\hat{p_{t}^{5}}}+\frac{C_{t}^{9}}{\hat{\tau}\hat{p_{t}^{9}}}+\frac{C_{t}^{10}}{\hat{\tau}\hat{p_{t}^{10}}}$$

Where $C_{t}^{l}$ is the catch in state *l* in time *t*, $\hat{p_{t}^{l}}$ is the estimated capture probability for state *l* in time *t*, and $\hat{\tau}$ is the estimate of the proportion of Colorado River fish in the Colorado River study site. Variances for abundance estimates were estimated using the Horvitz-Thompson estimator ([McDonald *et al.* 2005, p. 241](#_ENREF_2)). For adult estimates, we calculated the means and covariance matrix for the following quantitites $\hat{p_{t}^{4}}$, $\hat{p_{t}^{5}}$, $\hat{\tau}\hat{p_{t}^{9}}$, and $\hat{\tau}\hat{p_{t}^{10}}$ prior to applying the methods in McDonald *et al.* (2005) using exact equations for the covariance of a product of random variables ([Bohrnstedt & Goldberger 1969](#_ENREF_1)).

*Results:* The only state showing a consistent trend during the study was state 6, corresponding to juveniles in the Colorado River study sites. The abundance of this state increased steadily throughout the study, with most increases occurring between July and September (see part A of figure). Parts B and C of the below figure provide estimates of abundance for other states in October 2011 (the month in which abundance estimates for most states were most precise) and for the total adult population in September of each year.


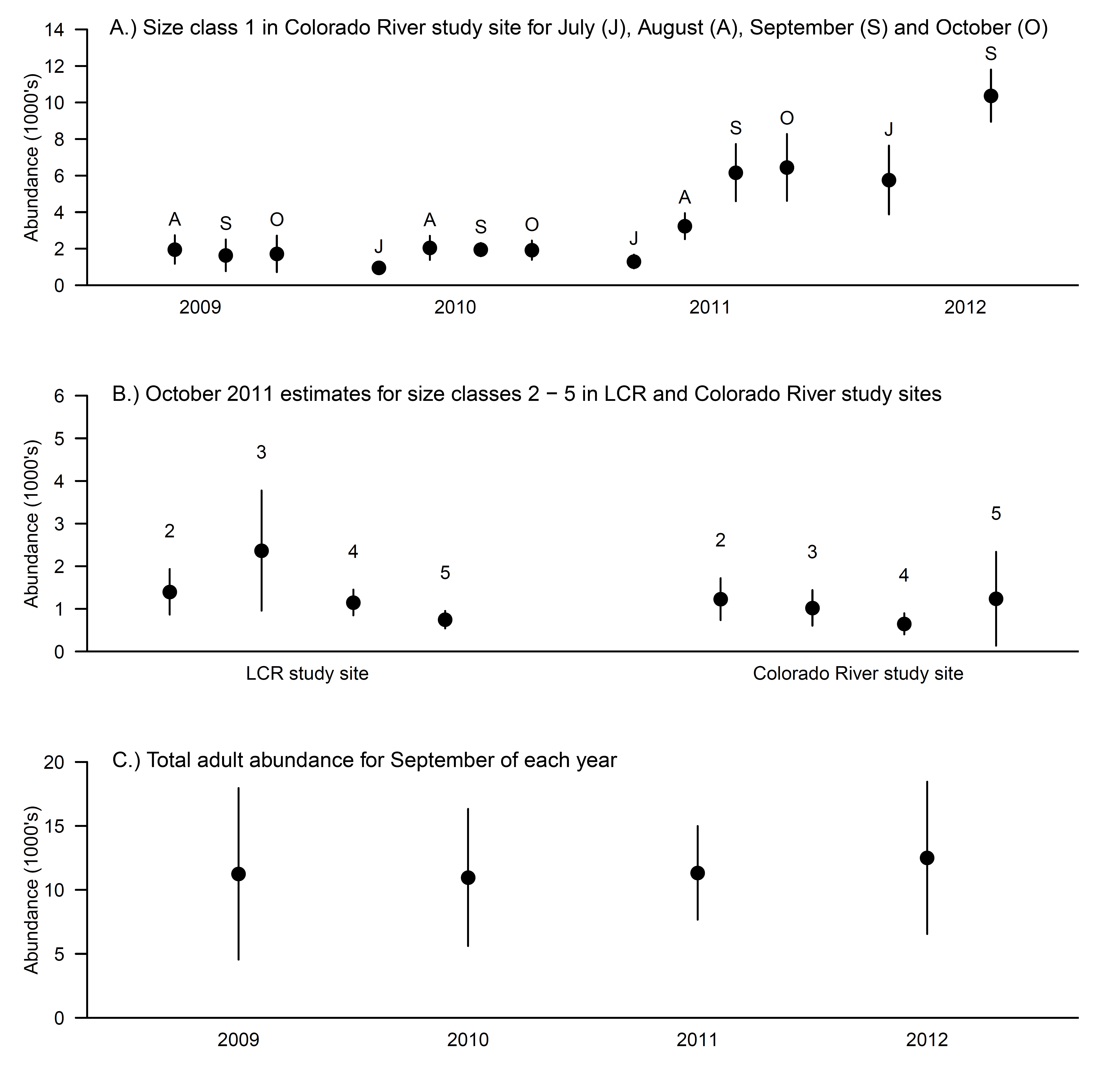


**Literature cited:**

Bohrnstedt, G.W. & Goldberger, A.S. (1969) On the Exact Covariance of Products of Random Variables. *Journal of the American Statistical Association,* **64,** 1439-1442.

McDonald, T.L., Amstrup, S.C., Regehr, E.V. & Manly, B.F. (2005) Examples. *Handbook of Capture-Recapture Analysis* (eds S.C. Amstrup, T.L. McDonald & B.F. Manly). Princeton University Press, Princeton.

Appendix D: GOF

Overall, observations from our best model matched predictions (see below). $\hat{c}$ for our best model was 1.686 indicating slight overdispersion, with much of the lack of fit coming from a few observed capture history that was predicted to be highly unlikely. For example, a single fish captured during August of 2009 in the Colorado River study site at 105 mm and recaptured in the LCR study site 380 meters from the confluence in September of 2012 at a length of 148 was responsible for increasing $\hat{c}$ from 1.515 to 1.686.


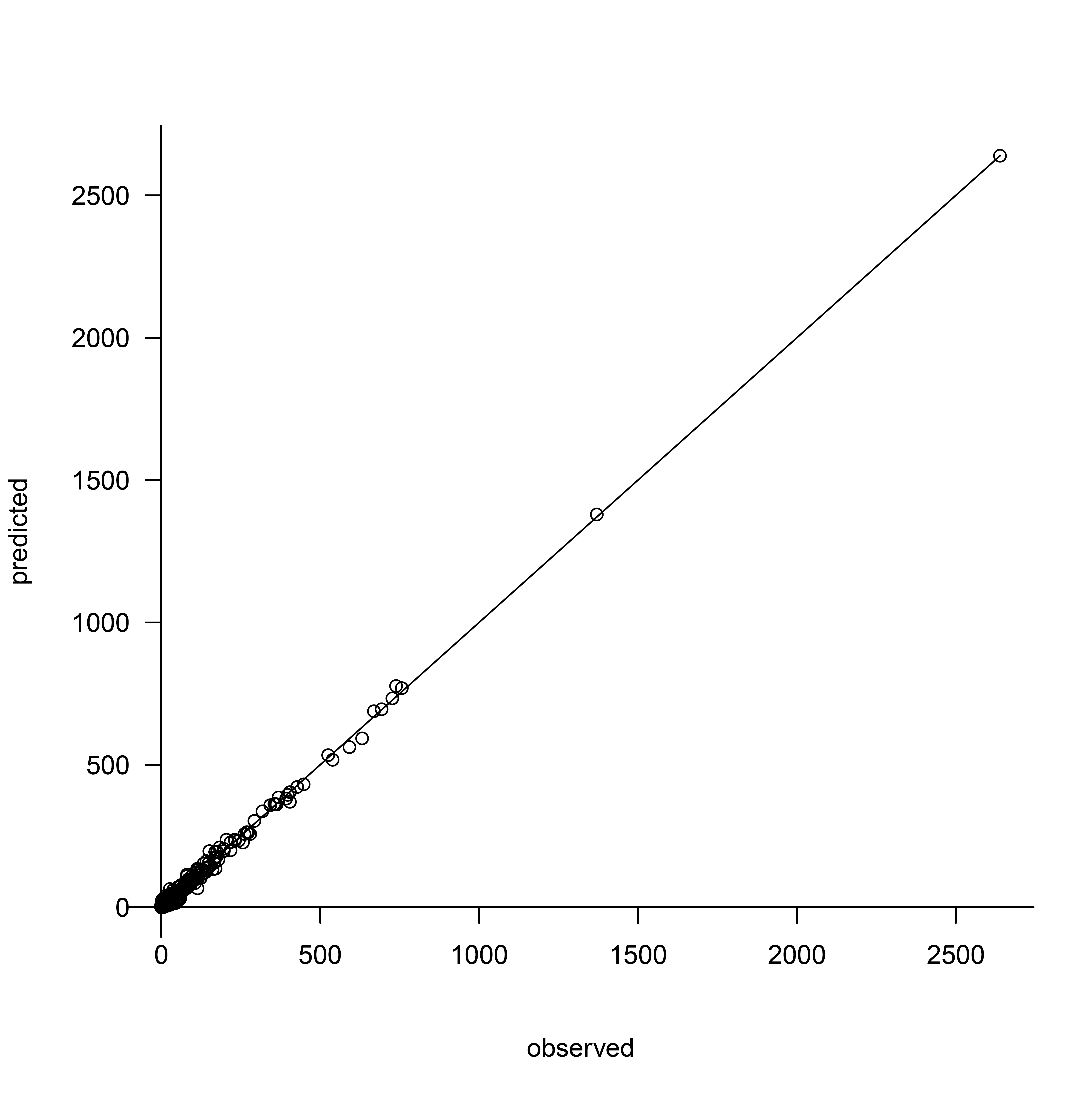

Supplement: Supplementary file 1 — Appendix S1. Annotated R code for fitting the global model described in the paper. Appendix S2. Tag retention study. Appendix S3. Abundance estimates. Appendix S4. GOF. [file ece30004-1006-sd1.docx]
